# Unmasking Early Renal Fibrosis in Polycystic Kidney Disease Using Noninvasive Precision Molecular MRI of Collagen

**DOI:** 10.64898/2026.07.19.739445

**Published:** 2026-07-20

**Authors:** Oluwabukola S. Bamishaye, Francis Akinlotan, Jingjuan Qiao, Phillip H. Chumley, Farzaneh Dorabadizare, Annye Bennett, Xiu Chen, Roye Ronald, Mandy J. Croyle, Khan Hekmatyar, Alton B. Farris, Anna G. Sorace, Harrison Kim, Jian-Xiong Wang, Hans E. Grossniklaus, Bradley K. Yoder, Michal Mrug, Jenny J. Yang

**Affiliations:** 1 Division of Hematology Oncology and Department of Medicine, University of Alabama Birmingham; Birmingham, AL, 30322, USA; 2 Center for Diagnostics and Therapeutics, Advanced Translational Imaging Facility, Department of Chemistry, Georgia State University; Atlanta, GA, 30303, USA.; 3 InLighta BioSciences, LLC; 5075 Lake Fjord Pass, Marietta, GA, 30068, USA; 4 Pathology Department, Emory University School of Medicine; Atlanta, GA, 30322, USA; 5 Eye Center, Emory University School of Medicine; Atlanta, GA 30322, USA; 6 Department of Radiology, University of Alabama Birmingham; Birmingham, AL, 30322, USA; 7 Division of Nephrology at the University of Alabama at Birmingham (UAB); Birmingham, AL, 35294, USA and Department of Veterans Affairs Medical Center, Birmingham, AL, 35233, USA; 8 Small Animal Imaging Shared Facility, O’Neal Comprehensive Cancer Center, 1700 University Blvd, Birmingham, AL, 35233, USA; 9 Comprehensive Cancer Center, The Ohio State University, Columbus, Ohio, 43210, USA; 10 Department of Cell, Development, and Integrative Biology, University of Alabama at Birmingham, Birmingham, AL 35294, USA

**Keywords:** ADPKD, Renal fibrosis, collagen-targeted MRI, chronic kidney disease, molecular imaging

## Abstract

Fibrotic remodeling of extracellular matrix is a central driver of autosomal dominant polycystic kidney disease (ADPKD) progression since early stages of the disease. Unfortunately, it cannot be assessed with currently available methodologies before irreversible structural and functional decline, creating a diagnostic blind spot that hinders accurate early risk stratification and management. Here, we report the development of Gd-hProCA32.Collagen, a collagen -targeted protein MRI contrast agent that enables precision molecular MRI (pMRI) of early fibrosis by directly imaging collagen type I deposition *in vivo* before conventional laboratory and imaging methods detect changes in kidneys and liver of *Pkhd*1^*PCK/PCK*^ (PCK) rats and *Pkd2* mutant mice. Gd-hProCA32.Collagen, used at 10-fold lower dose, outperformed the widely clinically used agent gadobutrol (Gadovist), detecting approximately 2.8-fold greater total renal cyst volume (~8,500 vs ~3,000 mm^3^, p<0.0001) and 1.5-fold higher total cyst count (~245 vs ~160, p<0.0001), with superior T1W and T2W kidney AUC (p<0.01 and p<0.001) and preferential sensitivity to small and medium cysts. Signal enhancement in kidneys and liver correlated strongly with histological collagen burden quantified by Sirius red staining, whereas Gadovist showed no meaningful correlation. Gd-hProCA32.Collagen also enabled *in vivo* visualization of previously undetectable changes resembling radial striations at sites of microcyst cluster-adjacent microfibrosis and sustained delayed MRI enhancement due to specific collagen binding. These results reveal a previously inaccessible subclinical fibrotic phase of cystic kidney and liver disease and establish collagen-targeted pMRI as a strategy for early noninvasive detection and spatial mapping of multi-organ extracellular matrix remodeling when conventional biomarkers remain non-discriminating.

## One-sentence summary

We report a first-in-class collagen-targeted MRI contrast agent with Precision molecular MRI that noninvasively reveals a previously inaccessible subclinical fibrotic phase of polycystic kidney disease across kidney and liver, overcoming limitations of current diagnostic methodology.

## Introduction

Chronic kidney disease (CKD) encompasses a spectrum of progressive disorders that impair renal structure and function^1^. CKD affects over 35 million Americans (~14% of adults), and most cases remain undetected until advanced stages, underscoring a critical gap in early diagnosis, risk assessment, and intervention. The landmark 2002 classification guidelines marked a pivotal shift in recognizing CKD as a global public health concern and advocated early-stage management by general practitioners and internists^1^. Early detection is vital since timely intervention can slow progression, reduce complication risk, and improve long-term outcomes^2^.

**Fig. 1.**
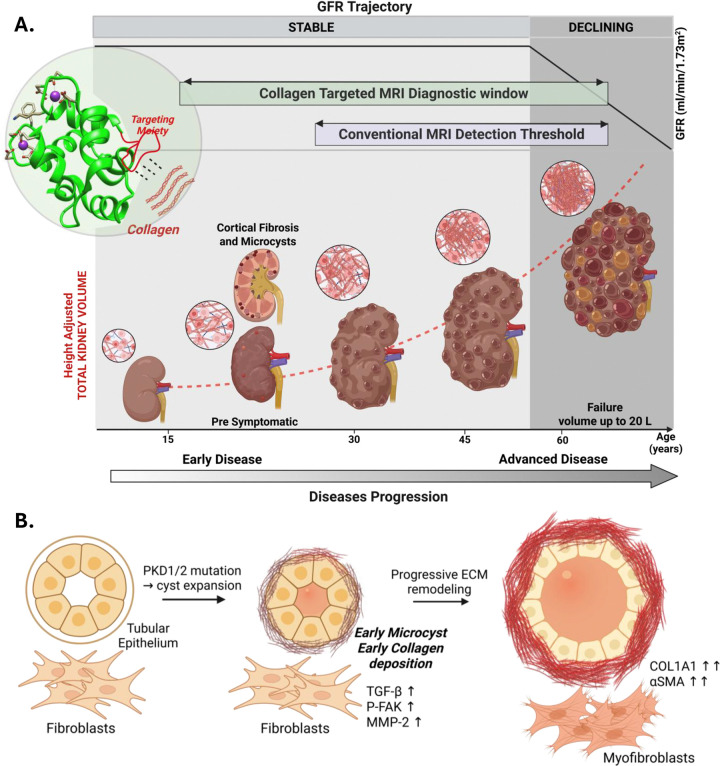
The role of collagen-targeted precision MRI in early detection of renal fibrosis during the pre-symptomatic window of ADPKD progression. (A) Schematic depicting the relationship of glomerular filtration rate (GFR) trajectory and height-adjusted total kidney volume (htTKV) in ADPKD progression. Kidney cross-sections illustrate progressive cystic and fibrotic burden from early pre-symptomatic disease (near normal volume in early age) through advancing disease and ensuing function impairment (increased kidney volume by middle age), and ESKD (large kidney volume in more advanced ages). GFR remains stable for decades despite ongoing structural injury, declining only in advanced disease, while htTKV (red dashed line) progressively grows since the functionally silent phase. Histological insets demonstrate progressive cortical fibrosis and microcyst formation culminating in near-total replacement of functional nephrons by collagen-rich cysts. The diagnostic window for collagen-targeted MRI may substantially precede the detection threshold of conventional MRI. Inset depicts the molecular architecture of Gd-hProCA32.Collagen comprising a protein-based targeting moiety (green helical structure) conjugated to gadolinium ions (purple spheres) that selectively binds extracellular collagen type I (red structure), the principal matrix component of renal fibrosis in ADPKD. (B) Schematic of pericystic fibrosis progression at the cellular level. PKD1 or PKD2 mutation drives tubular epithelial cyst expansion with early pericystic collagen deposition and activation of pro-fibrotic signaling, fibroblast-to-myofibroblast differentiation, and progressive extracellular matrix remodeling characterized by upregulation of collagen type I (COL1A1) expression. *(A) Created in BioRender. Yang lab, J. (2026)* https://BioRender.com/zmrnlkw *(B) Created in BioRender. Yang lab, J. (2026)* https://BioRender.com/snqle4e

The leading inherited cause of end-stage kidney disease (ESKD), and the fourth leading ESKD cause overall, is Autosomal Dominant Polycystic Kidney Disease (ADPKD). It is characterized by progressive renal and liver cyst expansion, interstitial fibrosis, and eventual loss of renal function ^3, 4^ (Fig. 1). Slowing cyst growth and fibrosis thus forms the critical foundation for future ADPKD care ^5–8^. Comprehensive risk stratification with longitudinal follow up essential for identifying patients at risk, selection of appropriate management strategies, and monitoring therapeutic responses ^9, 10^.

Renal fibrosis is among the central drivers of irreparable renal function loss common to most forms of CKDs, including ADPKD ^11^. Fibrosis is defined by remodeling of the extracellular matrix (ECM) that involves excessive type I collagen deposition. This leads to progressive disruption of renal architecture well before measurable functional decline ^12, 13, 14–16^. The consequences include irreversible loss of renal parenchyma, functional nephron depletion, and ultimately ESKD requiring dialysis or transplantation ^17, 18^. The pathogenesis of renal fibrosis is complex, attributed in part to persistent activation of myofibroblasts and ensuing abnormal (ECM) remodeling, resulting in progressive architectural distortion and irreversible loss of functional renal parenchyma ^19–25^. Notably, interstitial fibrosis is now recognized as an early and active disease driver rather than a late consequence of cyst expansion, preceding, accompanying, and potentially accelerating cystogenesis ^26, 27^ (Fig. 1). In addition to the renal involvement, hepatic manifestations affect more than 90% of ADPKD patients older than 35 years^28^. The hepatic manifestations may involve cholangiocyte proliferation, ductal plate malfunction, and progressive fibrosis, all of which may promote the formation of a collagen-rich microenvironment analogous to that observed in ADPKD kidneys ^29–31^.

Despite its central role in ADPKD progression and prediction of renal outcomes ^14–16, 32^, renal fibrosis cannot be directly assessed because a kidney biopsy is not practical for routine longitudinal assessment and it is even contraindicated in ADPKD ^32^. Therefore, the burden of renal fibrosis remains virtually unknown from early ADPKD till conventional renal function markers deteriorate from normal ranges due to substantial structural damage and functional tissue loss ^3, 33, 34^. In very early or mild disease, even the cyst volume-driven total kidney volume (TKV; often derived from T2-weighted MRI) may remain indiscriminatory, creating a diagnostic and prognostic blind spot. Intricacies of fibrosis assessment in cystic livers resemble those of cystic kidneys.

MRI has emerged as a promising noninvasive diagnostic and prognostic modality for ADPKD due to its favorable safety profile (e.g., lack of ionizing radiation), high soft-tissue contrast, and capability for multiparametric assessment ^35–37^. In particular, it has become the preferred method for assessment of TKV, a foundation of broadly used imaging classification for risk prediction assessment ^3, 38–40^. However, with respect to fibrosis detection, both routine and advanced MRI techniques (e.g., diffusion MRI, magnetization transfer, arterial spin labeling, BOLD MRI, and MR elastography) have insufficient molecular specificity for fibrosis and suffer from technical variability and lack of quantitative biomarker sensitivity ^38, 40–45^. Even these techniques cannot accurately detect microcysts (cysts <2–7 mm, MRI resolution limit) that likely contribute disproportionately to the ADPKD progression conductive microenvironment, given their overwhelming numerical predominance. Notably, smaller cysts have a disproportionately larger epithelial surface area relative to volume, conferring greater pro-fibrotic potential to promote abnormal ECM remodeling and function loss^46^. Even with current MRI methods, total cyst number and cyst parenchyma surface area offer superior prediction of the slope of kidney function decline and progression to kidney failure compared to TKV ^47^. In addition, dynamics of cyst volume changes over time is heterogeneous, with some cysts expanding rapidly, others remaining stable, and some regressing, leaving likely fibrotic remnants ^48, 49^. With current MRI techniques, detection of such processes remains technically challenging, clinically impractical, or both. Imaging approaches capable of directly detecting and quantifying microcysts and early-stage multi-organ fibrosis thus remain a major unmet need.

Current gadolinium-based contrast agents (GBCAs) have low relaxivity (r_1_ and r_2_ < 10 mM^−1^s^−1^), require the use of high doses for modest signal enhancement, raising potential safety concerns. Although newer macrocyclic GBCAs exhibit improved stability, current FDA, ACR, and NKF guidelines still recommend caution in patients with CKD, including ADPKD ^50–54^. Emerging evidence further suggests that repeated renal injury episodes may accelerate fibrosis progression and cyst-associated remodeling in PKD kidneys ^53, 55–57^. Because ADPKD kidneys exhibit altered tubular architecture, impaired flow dynamics, regional hypoxia, mitochondrial dysfunction, and increased susceptibility to injury ^58–61^, even transient contrast-mediated tubular stress may contribute to disease progression. Critically, these agents lack molecular specificity and cannot target collagen, precluding quantitative assessment of fibrosis ^62–67^. Efforts to develop targeted contrast agents by modifying small-molecule chelates have been limited by trade-offs between metal stability, relaxivity, and target binding, as well as limited expression of molecular biomarkers such as receptors and collagen (at sub-nM-μM range) ^37, 68–76^.

To overcome these limitations, we developed hProCA32.Collagen, a type I collagen-targeted, protein-based MRI contrast agent that enables direct molecular imaging of extracellular matrix remodeling. Building on our prior work in engineering high-relaxivity protein contrast agents ^77–79^, we hypothesized that this approach would enable high-sensitivity detection of early fibrosis and improve disease stratification at substantially lower doses than conventional agents. Here, we tested this hypothesis and also explored if collagen type I-targeted precision molecular MRI (pMRI) can reveal previously inaccessible subclinical fibrotic phases of ADPKD *in vivo* and if greater contrast enhancement quantitatively correlates with histological fibrosis. We also tested if this approach enables spatial mapping of early cortical fibrotic remodeling at stages when TKV and standard clinical biomarkers remain unchanged. We also studied if hProCA32.Collagen enables noninvasive, spatially resolved detection and staging of hepatic fibrosis in ADPKD. Favorable outcomes of these studies would indicate that pMRI may support comprehensive multi-organ disease early assessment within a single imaging framework, likely at reduced doses compared to conventional biochemical markers, which remain largely unchanged. Together, this technology would establish a foundation for a clinically translatable imaging platform for safe, early risk assessment, outcome prediction, disease progression, and therapeutic response monitoring in ADPKD.

## Results

### High Collagen Expression in Human ADPKD Establishes a Clinically Relevant Imaging Biomarker

To validate the clinical relevance of collagen type I as a potential imaging target for ADPKD progression, we quantitatively assessed collagen-positive area (CPA%) in human ADPKD kidneys and normal controls using histological sections stained with Sirius Red, a validated and reproducible quantitative measure of fibrotic burden.

**Figure 2.**
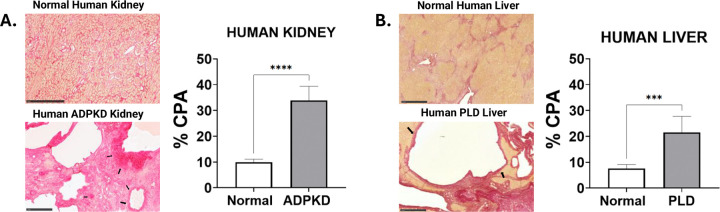
Histological validation of collagen overexpression in human ADPKD tissue. (A) Sirius Red staining of normal human kidney (top) and ADPKD kidney (bottom) demonstrates markedly elevated perilesional collagen deposition surrounding cysts in ADPKD (arrows) compared to normal kidney. Quantification of collagen-positive area (CPA) shows ~34% in ADPKD versus ~10% in normal kidney (p < 0.0001, ****), validating collagen as a clinically relevant imaging biomarker for renal fibrosis in ADPKD. (B) Sirius Red staining of normal human liver (top) and polycystic liver disease (PLD) tissue (bottom) shows increased pericystic collagen deposition around hepatic cysts (arrows) in PLD relative to normal liver. Quantification shows ~21% CPA in PLD versus ~8% in normal liver (p < 0.001, ***), supporting collagen as an imaging biomarker for hepatic fibrosis in PLD. Scale bars, 1 mm.

Human ADPKD kidneys exhibit markedly elevated collagen deposition localized to pericystic and interstitial regions, with CPA% significantly higher than in normal kidneys (~30% vs. ~9%; p < 0.0001) (Fig. 2A). The pronounced enrichment of collagen, especially in areas surrounding cysts, indicates active fibrotic remodeling associated with disease progression.

Parallel analysis of human ADPKD liver specimens reveals similarly elevated collagen deposition, with intense Sirius Red staining observed in the pericystic regions compared to normal liver tissue, with CPA% significantly higher than in normal liver (~21% vs. ~8%; p < 0.001) (Fig. 2B). These findings confirm that fibrosis and its enrichment in pericystic regions is a shared pathological feature across renal and hepatic ADPKD manifestations. Together with earlier reports^27, 80, 81^, these data support collagen type I as a robust, disease-relevant molecular biomarker for both renal and hepatic fibrosis in ADPKD, providing a strong biological and translational rationale for collagen-targeted precision MRI approaches such as hProCA32.Collagen. Importantly, the spatial localization and quantitative elevation of collagen deposition underscore the importance of noninvasive detection, staging, and longitudinal monitoring of multi-organ fibrotic progression in ADPKD patients. Moreover, the multi-organ distribution of collagen overexpression uniquely positions hProCA32.Collagen as a pan-organ imaging agent capable of simultaneously detecting and staging fibrotic burden across the kidneys and liver in a single imaging session, an unmet capability in current ADPKD clinical practice.

### Development and Characterization of hProCA32.Collagen as a Collagen-Targeted Protein Contrast Agent for Precision MRI of Renal and Liver Fibrosis

**Fig. 3.**
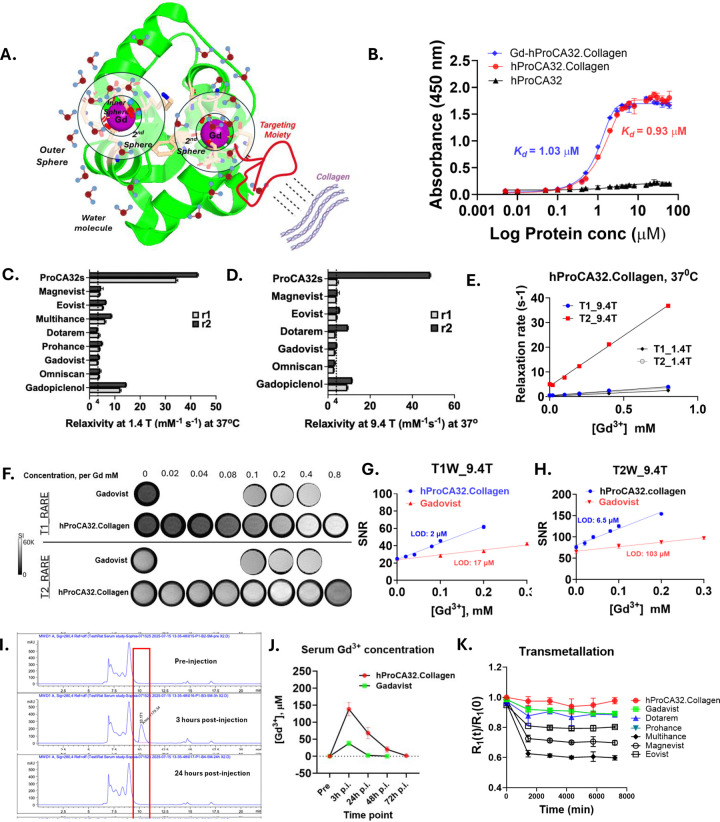
Biophysical characterization and safety validation of hProCA32.Collagen. (A) Schematic of hProCA32.Collagen showing the engineered protein scaffold (green ribbon), two gadolinium-binding sites (purple spheres) with inner, second, and outer sphere water coordination, and the collagen-targeting moiety (red loop) mediating specific binding to collagen I overexpressed in fibrotic and cancerous tissues. (B) Collagen binding affinity curves demonstrating high-affinity targeting (*K*_*d*_ = 1.03 μM and 0.93 μM for Gd-hProCA32.Collagen and hProCA32.Collagen, respectively); hProCA32 lacking the targeting moiety shows negligible binding, confirming collagen specificity is exclusively conferred by the targeting domain. (C and D) Longitudinal (r1) and transverse (r2) relaxivity of hProCA32.Collagen versus all clinically approved gadolinium-based contrast agents (GBCAs) at 1.4 T and 9.4 T (37°C), demonstrating superior dual-modal relaxivity. (E) Concentration-dependent T1 and T2 relaxation rates at 1.4 T and 9.4 T confirming linear contrast enhancement across both field strengths. (F) T1- and T2-weighted phantom images at 0–0.4 mM demonstrating superior signal enhancement versus Gadovist. (G and H) SNR quantification at 9.4 T revealing limits of detection of 2 μM and 6.5 μM for hProCA32.Collagen versus 17 μM and 103 μM for Gadovist (8-fold and 16-fold improvements, respectively). (I) Serum SEC-HPLC chromatograms at pre-injection, 3 hours, and 24 hours confirming progressive renal clearance and absence of systemic accumulation. (J) Serum Gd^3+^ concentration at multiple post-injection time points demonstrating return to baseline by 24 hours. (K) Transmetallation stability analysis confirming superior resistance to metal ion exchange versus six clinical agents over 8,000 minutes. Data points represent mean ± SD.

To enable molecularly specific, noninvasive detection of renal fibrosis in Autosomal Dominant Polycystic Kidney Disease (ADPKD), we developed hProCA32.Collagen, an engineered protein-based MRI contrast agent incorporating two Gd^3+^ chelation sites and a high-affinity collagen-targeting moiety designed to selectively bind type I collagen enriched in fibrotic tissues (Fig. 3A). The protein scaffold is rationally engineered to achieve high longitudinal (r_1_) and transverse (r_2_) relaxivity through coordinated contributions from inner-sphere, second-sphere, and outer-sphere water interactions, optimized water exchange kinetics, and an increased rotational correlation time (τR) ^77–79, 82, 83^.

Binding studies demonstrate that both apo- and gadolinium (Gd) -loaded hProCA32.Collagen retained strong affinity for type I collagen, with dissociation constants of *K*_*d*_ = 1.03 μM and *K*_*d*_ = 0.93 μM, respectively (Fig. 3B). In contrast, the non-targeted parental scaffold hProCA32 exhibits negligible binding across all tested concentrations (Fig. 3B), confirming the specificity conferred by the collagen-targeting domain. Critically, Collagen-binding affinity was maintained across rat serum, dog serum, mouse serum, and human plasma, with EC_50_ values ranging from 0.998 to 1.336 μM (Fig. S1), supporting the translational potential of hProCA32.Collagen across preclinical species and into human clinical applications.

hProCA32.Collagen demonstrated significantly superior longitudinal (r1) and transverse (r2) relaxivity compared to all clinically approved gadolinium-based contrast agents at both 1.4 T and 9.4 T at 37ºC (Fig. 3 C–E). At 9.4 T and 37°C, hProCA32.Collagen achieves an r_2_ of ~55 mM^−1^ s^−1^, exceeding the relaxivity of clinically approved GBCAs (r_2_ ≈ 4–5 mM^−1^ s^−1^) by >10-fold (Fig. 3D), highlighting its exceptional signal amplification capability at high field. To inform optimal MRI sequence selection for *in vivo* imaging at 9.4 T, theoretical signal intensity simulations were performed for hProCA32.Collagen and Gadovist across T1W_RARE and T2W_RARE sequences in liver and kidney tissue compartments (Fig. S2).

Inductively Coupled Plasma Optical Emission Spectroscopy (ICP-OES) analysis demonstrated that tissue Gd^3+^ concentrations in kidneys and liver of both PKD and control rats following administration of the imaging dose remained within the low physiological concentration range, peaking at approximately 210 μM (~0.21 mM) in PKD kidneys and approximately 170 μM in PKD liver at 24 hours post-injection, and substantially lower in control organ tissues (Fig. S3)

Phantom MRI studies further demonstrate superior sensitivity, with detectable signal enhancement at concentrations as low as 0.02 mM, approximately fivefold lower than Gadovist (0.1 mM) (Fig. 3F). Quantitative SNR analysis confirmed limits of detection of 2 μM versus 17 μM for Gadovist on T1W imaging (Fig. 3G), and 6.5 μM versus 103 μM for Gadovist on T2W imaging (Fig. 3H), representing 8.5-fold and 15.8-fold improvements in detection sensitivity on T1W and T2W sequences, respectively, consistent with the higher relaxivity of hProCA32.Collagen at both field strengths.

Serum pharmacokinetics was evaluated by Size-Exclusion Chromatography High-Performance Liquid Chromatography (SEC-HPLC) and ICP-OES analysis. SEC-HPLC chromatograms acquired at pre-injection, 3 hours, and 24 hours post-injection demonstrated progressive clearance of intact hProCA32.Collagen with complete elimination of the agent peak by 24 hours (Fig. 3I). Quantitative serum Gd^3+^ concentration analysis revealed distinct pharmacokinetic profiles between hProCA32.Collagen and Gadovist (Fig. 3J). While both agents showed peak serum Gd^3+^ concentrations at early post-injection timepoints, hProCA32.Collagen demonstrated a more sustained initial signal consistent with its larger molecular size and collagen-binding retention, followed by progressive clearance with Gd^3+^ levels returning to near-baseline by 48–72 hours post-injection. In contrast, Gadovist showed more rapid initial clearance consistent with its small molecular weight and lack of tissue binding. The return of serum Gd^3+^ to baseline confirms the absence of long-term gadolinium retention, a critical safety consideration given growing concerns regarding gadolinium deposition associated with repeated clinical GBCA administration. Organ-level biodistribution analysis by ICP-OES demonstrated preferential accumulation of hProCA32.Collagen in the kidneys and liver of PKD rats, the primary fibrosis-bearing organs in ADPKD, with peak Gd^3+^ concentrations at 24 hours post-injection, substantially exceeding those measured in control rats, directly reflecting the increased available collagen substrate in fibrotic kidney and liver tissues (Fig. S3). Substantial clearance from all organs was confirmed by 72 hours, with Gd^3+^ levels approaching baseline, confirming the absence of long-term gadolinium retention.

*In vivo* toxicological assessment confirmed that hProCA32.Collagen administration produced no statistically significant differences in organ weights across heart, liver, kidney, lung, or spleen compared to saline-treated controls (ns; p > 0.05), no gross morphological abnormalities in any harvested organ at 4 days post-injection, and no histopathological abnormalities, inflammatory infiltrates, necrosis, or tissue damage on H&E-stained sections of all five organs examined (Fig. S4), collectively confirming the biocompatibility and safety profile of hProCA32.Collagen at the imaging dose administered 4 days post-injection.

Importantly, transmetallation stability assays demonstrate enhanced resistance to endogenous metal ion competition, with hProCA32.Collagen outperforms six clinically approved GBCAs over >8000 minutes (Fig. 3K), supporting improved *in vivo* kinetic stability and safety for clinical translation. Collectively, these results establish hProCA32.Collagen as a high-affinity, collagen-specific protein MRI contrast agent with substantially enhanced relaxivity, sensitivity, and stability compared to conventional gadolinium-based agents, providing a strong mechanistic and biophysical foundation for its use in precision MRI (pMRI) of renal fibrosis in ADPKD.

### Defining the Diagnostic Gap in the PCK Rat Model

**Fig. 4.**
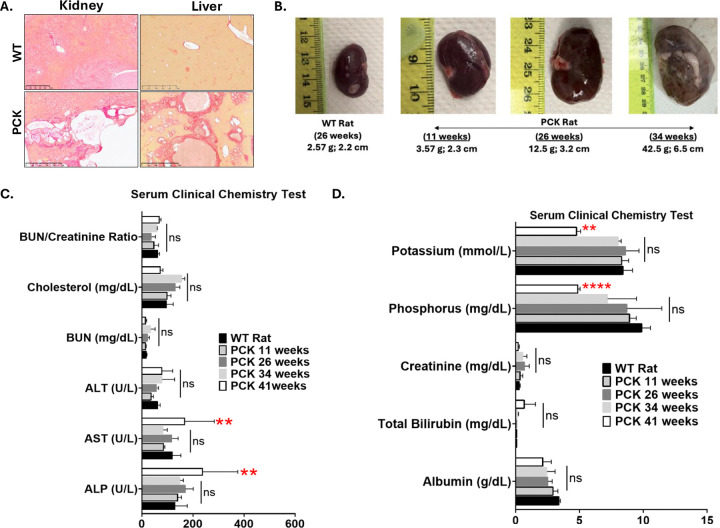
Histological, morphological, and serum clinical chemistry characterization of PCK rats across disease progression. (A) Representative Sirius Red-stained sections of kidney (left) and liver (right) from WT and PCK rats, demonstrating progressive cystic expansion and pericystic collagen deposition in PCK rats compared to normal tissue architecture in WT controls. (B) Representative gross morphological photographs of kidneys from wild-type (WT) rats at 26 weeks and PCK rats at 11, 26, and 34 weeks, illustrating progressive organ enlargement. (C) Serum hepatic and metabolic markers in WT and PCK rats at 11, 26, 34, and 41 weeks. ALP and AST showed significant elevations at advanced disease stages (p < 0.01), while ALT, BUN, cholesterol, and BUN/creatinine ratio remained within normal limits across all timepoints (ns), indicating preserved hepatic functional reserve despite extensive structural disease. (D) Serum electrolyte and renal function panel in WT and PCK rats at 11, 26, 34, and 41 weeks. Potassium was significantly decreased at advanced stages compared to WT (p < 0.01), and phosphorus showed a significant decrease at advanced disease stages (p < 0.0001), while creatinine, total bilirubin, and albumin remained unchanged (ns), supporting preserved renal filtration capacity despite progressive structural cystic burden. Data points represent mean ± SEM.

Among various ADPKD models, the PCK (*Pkhd1*^PCK^) rat ^48, 84, 85^, stands out as a central model used in seminal animal studies that preceded clinical testing of tolvaptan, so far the only ADPKD therapeutic approved by the FDA and other regulatory agencies. This model exhibits conserved ciliary localization defects, dysregulation of downstream signaling pathways, and robust phenotypic concordance with human disease, and has served as a translational platform in preclinical studies supporting therapies later evaluated in clinical trials. Since this model was not available on a standardized genetic background, we optimized this model by introducing it to a Sprague-Dawley (SD genetic background using rats from Charles River International Genetic Standardization Program, allowing the use of wild-type SD rats (WT) from this program as appropriate controls. Histopathological analysis demonstrates striking concordance between renal and hepatic fibrotic phenotypes in the PCK model, with Sirius Red staining revealing peri-cystic renal fibrosis (Fig. 4A, **left)** and concurrent congenital hepatic fibrosis with biliary involvement (Fig. 4A, **right)**, recapitulating the multi-organ fibrotic burden observed in human disease.

We further define a critical experimental window characterized by early cyst initiation and fibrotic remodeling that precedes overt functional decline (Fig. 4C–D). To interrogate the sensitivity of conventional biomarkers, we measured kidney sizes and comprehensive clinical chemistry profiling in PCK rats at 11, 26, 34, and 41 weeks of age, compared with age-matched WT SD controls, encompassing renal, hepatic, metabolic, and enzymatic parameters (Fig. 4).

Gross images of kidneys from wild-type (WT) rats at 26 weeks and PCK rats at 11, 26, 34, and 41 weeks of age demonstrate progressive kidney enlargement with disease progression across multiple disease stages (Fig. 4B). Analysis of primary renal function markers revealed that serum creatinine, BUN, and BUN/creatinine ratio showed no statistically significant differences between PCK and WT animals across any timepoint examined (all ns; p > 0.05; Table S1, Fig. 4C), consistent with preserved glomerular filtration function during the compensated phase of disease progression, a hallmark of the well-established dissociation between structural cystic burden and functional renal reserve in ADPKD. Despite this preservation of GFR-dependent markers, serum potassium was significantly decreased in PCK rats at advanced disease stages compared to WT controls (** p < 0.01), and phosphorus demonstrated a significant decrease at advanced stages (**** p < 0.0001; Fig. 4D), reflecting progressive tubular dysfunction and impaired electrolyte handling that parallels structural cystic burden. The preservation of creatinine, total bilirubin, and albumin across all timepoints (ns) further underscores the dissociation between electrolyte dysregulation and conventional GFR-dependent markers, highlighting the fundamental insensitivity of standard renal function tests to detect early-to-advanced structural tubular injury in PKD. Hepatic function markers, including ALT, BUN, cholesterol, and BUN/creatinine ratio, remained within normal reference ranges across all PCK groups and timepoints (all ns; p > 0.05; Table S1, Fig. 4C); however, AST and ALP demonstrated statistically significant elevations in PCK rats at advanced disease stages (** p < 0.01; Fig. 4C), potentially reflecting progressive biliary epithelial injury and periductal fibrosis associated with advancing hepatic cystic disease. Collectively, the fact that conventional clinical chemistry parameters failed to distinguish PCK from WT animals at early and moderately advanced stages (11–34 weeks), despite histologically confirmed bilateral cystogenesis, interstitial fibrosis, and progressive collagen deposition (Fig. 4A, Table S1), provides a compelling rationale for the development of sensitive, noninvasive molecular imaging approaches for *in vivo* fibrosis detection such as the collagen-targeted precision MRI with hProCA32.Collagen.

### PCK Rats Exhibit Progressive Renal Enlargement and Increased Kidney-to-Body Weight Ratio Despite Comparable Body and Liver Weight to Wild-Type Controls

**Fig. 5.**
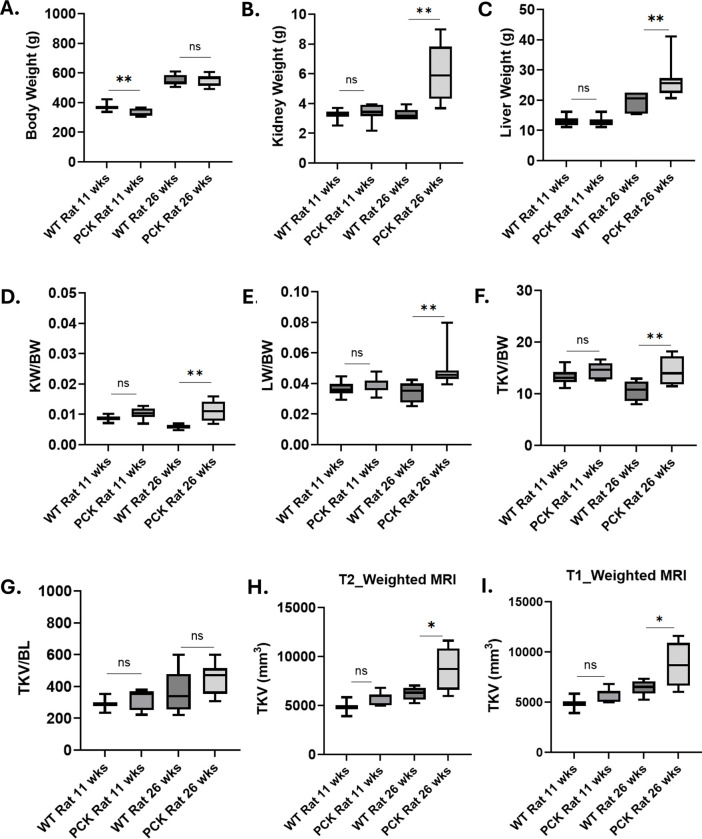
Morphometric and MRI-derived volumetric analysis of WT and PCK rats at early (11 weeks) and moderately advanced disease (26 weeks). (A–C) Body weight, kidney weight, and liver weight in wild-type (WT) and PCK rats at 11 and 26 weeks. No significant differences were observed at 11 weeks (ns); PCK rats at 26 weeks showed significantly lower body weight and significantly greater kidney and liver weights versus age-matched WT controls (p < 0.01), consistent with progressive cyst-driven organ enlargement and concurrent hepatic cystic involvement. (D and E) Kidney-to-body weight ratio (KW/BW) and liver-to-body weight ratio (LW/BW), demonstrating disproportionate organ enlargement relative to body mass in 26-week PCK rats (p < 0.01), with no significant differences at 11 weeks (ns). (F and G) Total kidney volume normalized to body weight (TKV/BW) and body length (TKV/BL). TKV/BW was significantly elevated in 26-week PCK rats (p < 0.01), while TKV/BL showed no significant differences at either timepoint (ns), indicating body length is a less sensitive normalizer for volumetric progression in this model. (H and I) MRI-derived TKV measured by T2W and T1W MRI, confirming significantly greater TKV in 26-week PCK rats versus WT controls (p < 0.05) with no significant differences at 11 weeks (ns), supporting multiparametric MRI as a reliable tool for longitudinal TKV assessment. Data are presented as box-and-whisker plots showing median, interquartile range, and full data range. Statistical comparisons between WT and PCK rats at each timepoint; *p < 0.05, **p < 0.01, ns = not significant.

Similar to prior reports^86, 87^, PCK rats at 26 weeks of age showed kidney and liver enlargement relative to age-matched WT controls. Specifically, PCK rats had significantly increased absolute kidney weight (KW; p < 0.01), kidney-to-body weight ratio (KW/BW; p < 0.01; for BW the p > 0.05), as well as TKV by T1 or T2 as absolute value or as ratio with BW (TKV and TKV/BW; p < 0.05 and p < 0.01, respectively); liver weight (LW; p < 0.01), and liver-to-body weight ratio were similarly increased (LW/BW; p < 0.01) (Fig. 5A–F). In contrast, no significant differences were observed between PCK and WT rats at 11 weeks for any of these parameters (all ns; p > 0.05), indicating that organ-level structural enlargement becomes statistically detectable at the moderately advanced disease. Total body weight was significantly lower in PCK rats at 11 weeks compared to WT controls (** p < 0.01), while body weights were comparable between groups at 26 weeks (ns, p = 0.834; Fig. 5A), indicating that the progressive kidney and liver enlargement observed at 26 weeks occurs independent of differences in overall body size.

In line with kidney weight measurements, MRI-based volumetric analysis revealed that T2-weighted MRI-derived TKV was significantly elevated in 26-week PCK rats compared to WT controls (p = 0.014), while no significant difference was detected at 11 weeks (p = 0.17; Fig. 5H), consistent with the organ weight findings and reflecting the emergence of macroscopic cystic expansion at moderately advanced disease. T2-weighted imaging is the conventional standard for TKV assessment in PKD, as the high water content of cystic fluid produces strong T2 hyperintensity, enabling clear delineation of cyst boundaries from surrounding parenchyma and facilitating accurate volumetric segmentation. T1-weighted MRI-derived TKV demonstrated a similar directional trend of progressive increase in PCK rats at 26 weeks, reaching statistical significance (p < 0.05; Fig. 5I), confirming concordance between T1W and T2W volumetric measurements and validating multiparametric MRI as a reliable tool for longitudinal TKV assessment in this model. TKV normalized to body length (TKV/BL) showed no statistically significant differences between PCK and WT rats at either 11 weeks (p = 0.41) or 26 weeks (p = 0.18; both ns), suggesting that body length is a less sensitive normalizer for detecting volumetric progression in this model compared to absolute TKV.

Importantly, while MRI-derived TKV successfully captured macroscopic kidney enlargement at 26 weeks, this volumetric metric provided no information on the underlying molecular and microstructural processes driving disease progression, specifically the fibrotic remodeling, collagen deposition, and pericystic matrix expansion that histological analysis confirms are already present at 11 weeks (Fig. 4A), a timepoint at which TKV and TKW-based indices remain statistically indistinguishable from WT controls. This fundamental limitation of gross anatomical metrics, such as TKV, their inability to detect or quantify the biologically critical early fibrotic transition that precedes macroscopic organ enlargement, indicates the need for molecularly targeted imaging approaches capable of directly quantifying collagen deposition and fibrotic activity *in vivo*, providing a compelling imaging-based rationale for the collagen-targeted precision MRI strategy evaluated in this study.

### hProCA32.Collagen Enables Superior Cyst-Size-Dependent MRI Enhancement, Region-Specific Fibrosis Mapping, and Histological Validation in the PCK Rat Kidney

**Fig. 6.**
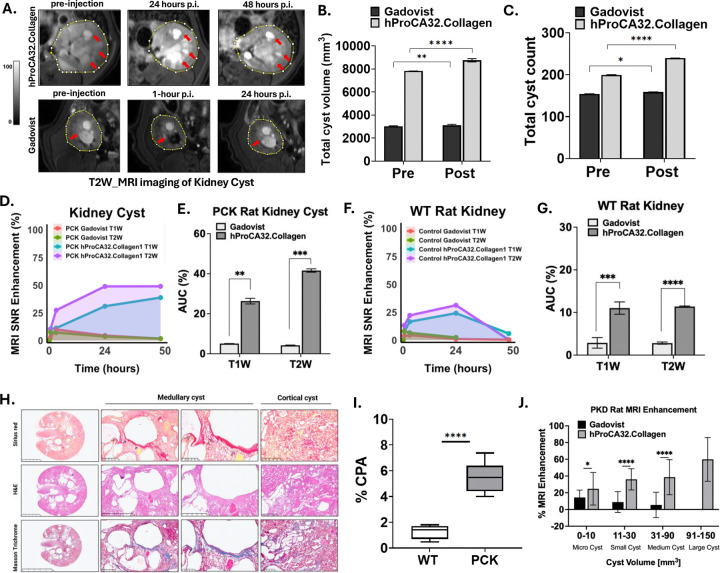
Collagen-targeted MRI enables superior detection and staging of renal cystic and fibrotic burden in PCK rats compared to Gadovist. (A) Representative SNR Map from T2-weighted MRI of PCK rat kidneys (yellow dashed ROI) at pre-injection, 24 hours, and 48 hours post-injection with hProCA32.Collagen (top) and pre-injection, 1 hour, and 24 hours post-injection with Gadovist (bottom), demonstrating markedly superior and sustained signal enhancement with hProCA32.Collagen. (B and C) Total cyst volume and total cyst count detected pre- and post-injection, showing significantly greater cyst volume (~8,500 vs. ~3,000 mm^3^, p < 0.0001) and cyst count (~245 vs. ~160, p < 0.0001) with hProCA32.Collagen versus Gadovist. (D and E) MRI SNR enhancement time-courses and AUC in PCK rat kidneys demonstrating significantly superior T1W and T2W enhancement with hProCA32.Collagen over 48 hours (p < 0.01 and p < 0.001, respectively). (F and G) SNR enhancement and AUC in WT rat kidneys confirming significantly elevated T1W and T2W enhancement with hProCA32.Collagen versus Gadovist (p < 0.001 and p < 0.0001, respectively). (H) Representative Sirius Red, Masson’s Trichrome, and H&E-stained sections of medullary and cortical cysts from PCK rat kidneys demonstrating pericystic collagen deposition. (I) Collagen proportionate area (CPA) quantification confirming significantly greater fibrosis burden in PCK versus WT rats (p < 0.0001). (J) MRI enhancement stratified by cyst volume (0–150 mm^3^), demonstrating significantly superior detection of small and medium cysts with hProCA32.Collagen versus Gadovist (p < 0.0001). Data are mean ± SEM.

We systematically evaluated the *in vivo* molecular imaging performance of hProCA32.Collagen for detecting cystic and fibrotic remodeling in kidneys using longitudinal MRI acquired pre-injection and following intravenous administration of hProCA32.Collagen or Gadovist in PCK and WT rats (23–26 weeks of age) (Fig S5). At an injection dose of 0.010 mmol/kg (10-fold lower than Gadovist), hProCA32.Collagen produced ~40% and 50% sustained signal-to-noise ratio (SNR) enhancement for both T1- and T2-weighted MRI within PCK kidney cysts, respectively. In contrast, Gadovist produced only minimal and transient SNR enhancement peaking at 1 hour post-injection and returning toward baseline by 24 hours (Fig. 6A, D). Quantitative AUC analysis confirmed significantly superior T1W (p < 0.01) and T2W (p < 0.001) enhancement for hProCA32.Collagen relative to Gadovist in PCK kidney cysts, with mean AUC values of 26.3% (T1W) and 41.6% (T2W) for hProCA32.Collagen compared to 5.1% (T1W) and 4.2% (T2W) for Gadovist (Fig. 6E). hProCA32.Collagen enhancement persisted through 48 hours post-injection, consistent with sustained collagen-specific molecular binding at sites of active fibrotic remodeling, in contrast to the rapid washout observed with Gadovist (Fig. 6D; Fig. S6A). Quantitative T2 relaxometry further confirmed the specificity and tissue retention of hProCA32.Collagen: ΔR2 in PCK rat kidney cysts increased progressively from 5.6 s^−1^ at 30 minutes to 14.8 s^−1^ at 48 hours post-injection, while Gadovist produced only minimal and declining ΔR2 values across all timepoints (Fig. S8). In WT rat kidneys, hProCA32.Collagen produced moderate T1W (p < 0.001) and T2W (p < 0.0001) enhancement peaking at 24 hours and returning to baseline by 48 hours due to renal clearance, with no cysts detected in control animals (Fig. 6F, G). Histological characterization of renal fibrosis and cystic architecture across cortical and medullary regions is shown in Fig. 6H.

hProCA32.Collagen increased total detectable cyst volume (p < 0.0001) and cyst count (p < 0.0001) from pre- to post-injection in PCK rats, demonstrating its ability to unmask previously undetectable microcysts (Fig. 6B, C). In contrast, Gadovist produced only modest increases in cyst volume (p < 0.05) and cyst count (p < 0.01). Collectively, hProCA32.Collagen increased the total number of detectable cysts by 21% compared to only 2.6% with Gadovist (p < 0.001). Cyst-size–stratified analysis revealed consistent and size-independent superiority of hProCA32.Collagen over Gadovist across all cyst volume categories: microcysts (0–10 mm^3^, p < 0.05), small (11–30 mm^3^, p < 0.0001), medium (31–90 mm^3^, p < 0.0001), and large cysts (91–150 mm^3^, p < 0.0001) (Fig. 6I, J), establishing broad cyst detection capability across the full size spectrum. Individual cyst-level analysis of right and left kidneys in PCK rats demonstrated that hProCA32.Collagen produced consistently positive and substantial percent MRI enhancement across all individually segmented cysts regardless of cyst volume, demonstrating uniform collagen-targeted signal amplification (Fig. S7A, B). In contrast, Gadovist produced highly variable and frequently negative percent MRI enhancement values across individual cysts in both kidneys, with no consistent relationship between cyst volume and signal enhancement (Fig. S7C, D), confirming the nonspecific, passive distribution behavior of conventional GBCAs at the individual cyst level. In addition, post-injection MRI enhancement with hProCA32.Collagen correlated positively with cyst volume (r = 0.37), whereas Gadovist showed no meaningful correlation (r = 0.09) (Fig. S6B), further confirming collagen-targeted molecular binding rather than nonspecific accumulation. ROC curve analysis of T2W SNR enhancement demonstrated perfect diagnostic accuracy for Gd-hProCA32.Collagen in distinguishing PCK fibrotic from WT kidneys (AUC = 1.000, 95% CI: 1.000–1.000, p = 0.007) and from PCK rats with Gadovist at its peak enhancement timepoint (AUC = 1.000, 95% CI: 1.000–1.000, p = 0.003), achieving 100% sensitivity and 100% specificity by Youden’s J index (Fig. S10A, B). Gadovist showed non-significant discrimination between PCK and WT kidneys even at peak enhancement (AUC = 0.933, p = 0.053) (Fig. S10C), confirming the superior diagnostic accuracy of collagen-targeted MRI over the clinical standard agent.

### hProCA32.Collagen Enables Precision Mapping of Renal Fibrotic Striation and Focal Collagen Deposition with Quantitative MRI–Histology Correlation

**Fig. 7.**
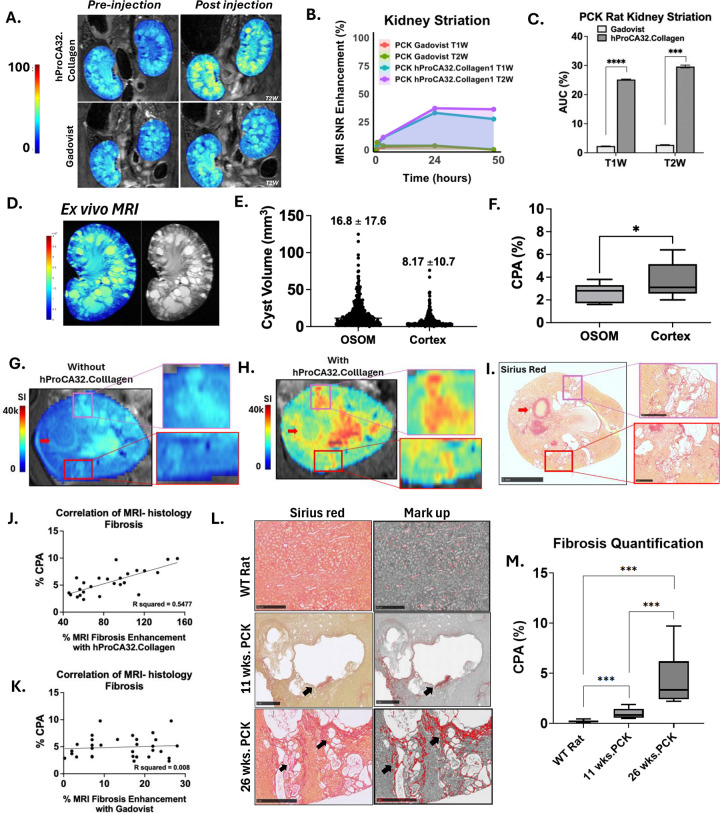
Collagen-targeted MRI detects pericystic renal fibrosis and cortical fibrotic microenvironments with histological validation in PCK rats. (A) Representative T2-weighted MRI SNR maps of PCK rat kidneys pre- and post-injection with hProCA32.Collagen (top row) and Gadovist (bottom row), demonstrating sustained and progressive signal enhancement within radially organized fibrotic striation regions with hProCA32.Collagen, while Gadovist produced no appreciable enhancement at equivalent timepoints. (B and C) MRI SNR enhancement time-courses and AUC for kidney striation regions in PCK rats, demonstrating T1W and T2W enhancement with hProCA32.Collagen versus Gadovist (p < 0.0001 and p < 0.001, respectively). (D) Ex vivo T2W MRI signal intensity map of PCK rat kidneys confirming sustained hProCA32.Collagen enhancement with spatial correspondence to cystic architecture. (E) Cyst volume distribution in outer strip of medulla (OSOM; 16.8 ± 17.6 mm^3^) and cortex (8.17 ± 10.7 mm^3^) compartments. (F) CPA quantification demonstrating significantly greater cortical fibrosis versus OSOM (p < 0.05). (G and H) Representative SI maps of cortical fibrotic microenvironments without and with hProCA32.Collagen, with magnified insets demonstrating focal signal enhancement in collagen-rich pericystic regions undetectable without the agent. (I) Corresponding Sirius Red histology confirming collagen deposition at MRI-enhanced cortical regions. (J and K) Linear regression of MRI fibrosis enhancement versus CPA demonstrating strong correlation with hProCA32.Collagen (R^2^ = 0.55) and no correlation with Gadovist (R^2^ = 0.008). (L) Representative Sirius Red staining and digital markup of WT rat and PCK rat kidneys at 11 and 26 weeks. (M) CPA quantification confirming progressive fibrosis from WT to 11-week and 26-week PCK rats (p < 0.001). Data are mean ± SEM.

Gd-hProCA32.Collagen enabled, to our knowledge, the first *in vivo* visualization of radially organized fibrotic striations associated with clusters of cysts and microcysts, manifesting on MRI as ray-like signal projections extending from the medulla toward the renal capsule (Fig. 7A). Representative pre- and post-injection T2-weighted MRI images demonstrate that hProCA32.Collagen produced sustained and progressive signal enhancement within these striated fibrotic regions, while Gadovist produced no appreciable enhancement at equivalent timepoints (Fig. 7A). These striated fibrotic regions exhibited sustained and progressive SNR enhancement on both T1- and T2-weighted imaging, peaking at 24 hours and persisting through 48 hours post-injection, while Gadovist produced only minimal and transient signal change across all timepoints (Fig. 7B). Quantitative AUC analysis confirmed striation enhancement with hProCA32.Collagen compared to Gadovist on both T1W (p < 0.0001) and T2W (p < 0.001) sequences (Fig. 7C), with mean AUC values of 26.3% (T1W) and 41.6% (T2W) for hProCA32.Collagen compared to 5.1% (T1W) and 4.2% (T2W) for Gadovist, representing approximately 5–6-fold greater enhancement consistent with high-affinity collagen-targeted molecular retention at sites of active fibrotic remodeling.

*Ex vivo* high-resolution MRI colormaps of excised PCK rat kidneys confirmed the spatial localization of fibrotic striation regions and heterogeneous cyst distribution, revealing region-specific collagen deposition patterns that closely recapitulate the striated fibrosis architecture observed histologically (Fig. 7D). Region-specific quantification demonstrated that OSOM cysts were substantially larger than cortical cysts (16.8 ± 17.6 mm^3^ vs. 8.17 ± 10.7 mm^3^; Fig. 7E), while histological CPA% quantification confirmed significantly greater fibrotic burden in the cortex relative to the OSOM (p < 0.05; Fig. 7F), consistent with fibrosis scoring results (p < 0.0001; Fig. S11C), establishing that OSOM-predominant cyst expansion and cortex-predominant fibrosis represent spatially distinct yet complementary pathological processes.

Direct co-registration of *in vivo* MRI with histopathology revealed discrete focal fibrotic striation lesions that were substantially more conspicuous on hProCA32.Collagen-enhanced images (Fig. 7H) compared to images acquired without the agent (Fig. 7G), demonstrating that collagen-targeted contrast enhancement significantly improves the detectability of focal pericystic fibrotic regions that are otherwise poorly delineated on unenhanced MRI. Sirius Red whole kidney sections (Fig. 7I) confirmed the overall distribution of focal collagen deposition across cortical and medullary compartments, broadly consistent with the regions of hProCA32.Collagen-enhanced signal, though spatial correspondence between MRI enhancement foci and histological collagen deposition is regional rather than exact, reflecting inherent differences in resolution and sectioning plane between *in vivo* MRI and histology.

Quantitative pMRI–histology correlation demonstrated a positive association between hProCA32.Collagen signal enhancement and histological CPA% (R^2^ = 0.5477; Fig. 7J), whereas Gadovist signal showed no meaningful correlation with fibrosis burden (R^2^ = 0.008; Fig. 7K), confirming that hProCA32.Collagen enhancement quantitatively and specifically reports collagen deposition at sites of active renal fibrosis. Histological validation using Sirius Red staining and digital markup confirmed stage-dependent progression of collagen deposition, with minimal fibrosis in WT kidneys, early periglomerular and peritubular collagen accumulation at 11 weeks in PCK rats, and extensive fibrotic bridging with radially organized striations at 26 weeks (Fig. 7L). Region-resolved quantitative CPA% analysis demonstrated significant and progressive fibrosis accumulation across PCK disease stages, significantly elevated at 11 weeks versus WT (p < 0.001), further increased at 26 weeks versus 11 weeks (p < 0.001), and elevated at 26 weeks versus WT (p < 0.001), establishing fibrosis as a continuous and histologically measurable process throughout PCK disease progression (Fig. 7M). Compartment-specific histological analysis further confirmed that medullary cysts are substantially larger than cortical cysts (17.6 ± 21.0 mm^3^ vs. 5.60 ± 6.12 mm^3^; Fig. S11B), while cortical fibrosis burden significantly exceeds that of the OSOM (p < 0.0001; Fig. S11C), with wild-type kidney sections showing preserved normal renal architecture and minimal collagen deposition across all staining methods (Fig. S11D).

Taken together, these results establish that hProCA32.Collagen noninvasively resolves two spatially distinct yet complementary pathological processes: early cortical microfibrosis and OSOM-dominant cyst enlargement. Notably, hProCA32.Collagen enables detection and quantification of early cortical fibrotic remodeling at disease stages when TKV and conventional biochemical markers remain largely unchanged. This positions hProCA32.Collagen as a sensitive molecular imaging biomarker for early disease stratification and longitudinal progression monitoring in ADPKD.

### hProCA32.Collagen Enables Non-Invasive Detection and Staging of Hepatic Fibrosis and Liver Cysts in PCK Rats

Given the well-established multi-organ burden of ADPKD, which frequently involves progressive hepatic cystogenesis with pericystic (and in some cases periportal) fibrosis in parallel with renal disease, we investigated whether hProCA32.Collagen can enable concurrent staging of hepatic fibrosis in the same imaging session used for renal assessment in the studied ADPKD animal models.

**Fig. 8.**
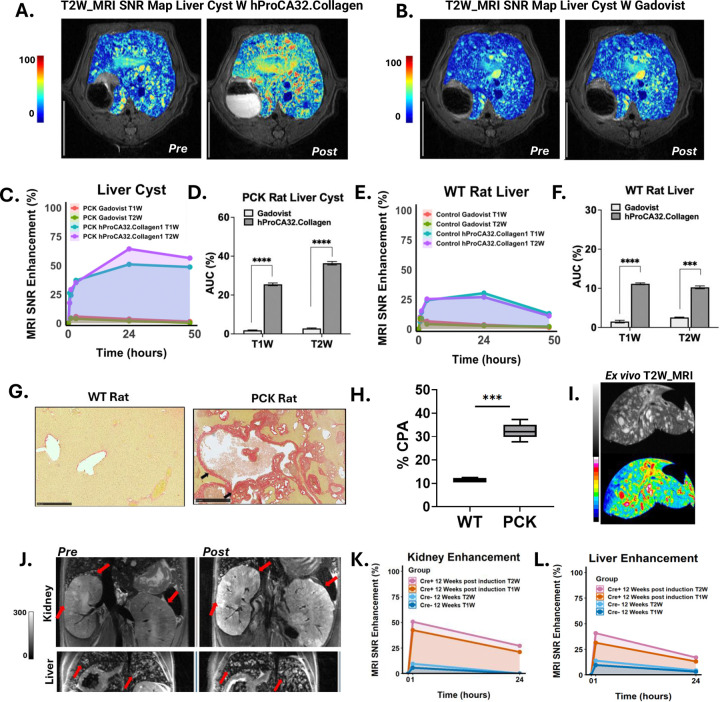
Collagen-targeted MRI enables superior detection of hepatic cystic and fibrotic burden in PCK rats and translational validation in a Cre-inducible ADPKD mouse model. (A and B) Representative T2W MRI SNR maps of PCK rat liver at pre- and post-injection with hProCA32.Collagen (A) and Gadovist (B), demonstrating markedly superior and sustained hepatic signal enhancement with hProCA32.Collagen versus minimal enhancement with Gadovist. (C and D) MRI SNR enhancement time-courses and AUC for PCK rat liver cysts demonstrating significantly superior T1W and T2W enhancement with hProCA32.Collagen over 48 hours (p < 0.0001 for both sequences). (E and F) SNR enhancement and AUC in WT rat liver confirming significantly elevated enhancement with hProCA32.Collagen versus Gadovist (p < 0.0001 and p < 0.001), with rapid clearance in the absence of fibrotic disease. (G) Representative Sirius Red sections from WT (left) and PCK (right) rat liver demonstrating minimal collagen deposition in WT versus extensive pericystic and periportal fibrosis in PCK rats (black arrows). (H) CPA quantification confirming significantly greater hepatic fibrosis in PCK versus WT rats (~33% vs. ~11%, p < 0.001). (I) *Ex vivo* T2W MRI signal intensity map of PCK rat liver demonstrating heterogeneous enhancement spatially corresponding to cystic and fibrotic regions. (J) Representative T2W MRI of kidney (top) and liver (bottom) in 12-week Cre-inducible ADPKD mice pre- and post-injection with hProCA32.Collagen, with red arrows indicating regions of focal enhancement. (K and L) MRI SNR enhancement time-courses for kidney and liver in Cre+ (12 weeks post-induction) versus Cre− mice, demonstrating significantly greater T1W and T2W enhancement in Cre+ mice across both organs. Data are mean ± SEM.

Following intravenous administration of hProCA32.Collagen, T2-weighted MRI signal-to-noise ratio (SNR) maps of PCK rat liver cysts demonstrated prolonged signal enhancement compared to Gadovist (Fig. 8A–B). hProCA32.Collagen produced sustained and progressive SNR enhancement in PCK rat liver cysts on both T1- and T2-weighted imaging, remaining significantly elevated across all timepoints up to 48 hours post-injection, while Gadovist showed only minimal and transient enhancement that rapidly cleared by 24 hours post-injection (Fig. 8C). Quantitative AUC analysis confirmed T1W and T2W enhancement by hProCA32.Collagen compared to Gadovist in PCK rat liver cysts (p < 0.0001), demonstrating collagen-targeted retention within fibrotic pericystic tissue (Fig. 8D).

In the WT rat liver, hProCA32.Collagen produced moderate signal enhancement on both T1- and T2-weighted imaging that was higher compared to Gadovist (T1W: p < 0.0001; T2W: p < 0.001), yet substantially lower than that observed in fibrotic PCK rat liver across all timepoints (Fig. 8E and F). This differential enhancement between PCK and WT rat liver supports the molecular specificity of hProCA32.Collagen for collagen-rich fibrotic tissue, with reduced enhancement in healthy liver reflecting the absence of abundant periportal and pericystic collagen deposition.

*Ex vivo* T2-weighted MRI with corresponding colorimetric SNR color map analysis of PCK rat liver demonstrated heterogeneous signal distribution with regional variations in fibrotic burden and cyst distribution (Fig. 8I), providing high-resolution spatial mapping of hepatic cysts and fibrotic compartments that closely correspond to histological findings. Sirius Red histological staining confirmed significantly elevated periportal and pericystic collagen deposition in the PCK rat liver compared to normal WT rat liver architecture (Fig. 8G). Quantitative analysis of collagen-positive area (CPA%) demonstrated significantly higher fibrosis burden in PCK fibrotic liver (~30%) compared to normal liver (~10%; p < 0.001), providing direct histological corroboration of the molecularly targeted MRI signal observed *in vivo* (Fig. 8H).

Notably, despite progressive hepatic cystogenesis and biliary fibrosis in PCK rats, conventional liver function markers, including ALT, AST, GGT, albumin, total protein, and total bilirubin, remained comparable to those of age-matched WT control rats at all disease stages (all p = ns) (Fig. 4C–D). This dissociation between structural fibrotic burden, detectable by hProCA32.Collagen-enhanced MRI and preserved conventional biochemical markers underscore the diagnostic blind spot of current clinical tools and highlight the unique capability of collagen-targeted molecular MRI to detect and stage hepatic fibrosis before functional decompensation becomes apparent. Collectively, these findings establish a strong foundation for hProCA32.Collagen as a sensitive and molecularly specific imaging agent for noninvasive detection and spatial staging of hepatic fibrosis in ADPKD, extending precision molecular MRI from renal to hepatic disease involvement and enabling comprehensive multi-organ fibrosis assessment within a single imaging session.

### hProCA32.Collagen enables the Non-Invasive Detection of Renal and Hepatic Fibrosis and Cysts in PKD2 mouse model

To provide further mechanistic validation across genetically distinct PKD backgrounds, we conducted similar analysis using the Pkd2 conditional mutant mouse. The conditional Pkd2 mouse exhibits distinct rates of cyst progression and fibrosis, enabling temporal analysis of early disease remodeling before overt renal dysfunction ^48, 84, 85^ (Fig. 1A). Cre^+^ mice demonstrated progressive and dramatic renal enlargement confirmed by significantly elevated kidney weight and exponentially increasing TKV at our analysis timepoints between 5 and 20 weeks post-induction, while body weight remained comparable to Cre^−^ controls until late-stage disease (Fig. S12A–C). hProCA32.Collagen administration produced significantly higher and more sustained SNR enhancement in both kidney and liver of Cre^+^ mice compared to Cre^−^ controls at 1 and 24 hours post-injection on both T1- and T2-weighted imaging, with marked signal enhancement visually confirmed by pre- and post-injection MRI (Fig. 8J–L), consistent with the significantly elevated collagen deposition confirmed histologically by Sirius Red staining in both organs (CPA: kidney ~1.5%, liver ~1%; Fig. S12E–F).

### High safety profile of Gd-hProCA32.collagen

The administration of Gd-hProCA32.collagen at > 10-fold lower Gd dose injection than approved GBCA such as Gadovist did not result in any overt cytotoxicity (Fig. S4) and tissue toxicity. We also demonstrate that the Gd injected is excreted from the animal with insignificant residual metal ions in key tissues such as brain, bone, kidney, liver, and lung (Fig. S3), which is likely due to the strong metal-binding affinity and selectivity and kinetic stability of the developed agent (Fig 2J–K). Thus, Gd-hProCA32.collagen is likely to minimize toxicity concerns associated with approved GBCA for renal diseases.

## Discussion

In this study, we show, to our knowledge, the first *in vivo* application of a collagen-targeted, protein-based precision MRI platform for simultaneous molecular detection enabling spatial mapping and staging of renal and hepatic fibrosis in ADPKD models. hProCA32.Collagen enables visualization of subclinical extracellular matrix remodeling, pathological collagen deposition, and cyst- associated cortical fibrosis in very early disease stages when conventional functional biomarkers and approved GBCA remain indiscriminatory. In contrast, hProCA32.Collagen detects histologically validated collagen depositions at timepoints when all biochemical markers are within normal ranges and cannot be determined by conventional T2W methods. These findings redefine collagen-targeted molecular MRI as a mechanistically grounded and quantitatively validated imaging paradigm, addressing longstanding limitations in ADPKD severity and prognosis assessment in early, mild, or atypical presentations.

The superior imaging performance of hProCA32.Collagen relative to Gadovist reflects a fundamental mechanistic distinction between molecularly targeted contrast retention and passive extracellular distribution. hProCA32.Collagen produces up to ~5-fold greater and temporally sustained enhancement across all cyst size categories, from microcysts (0.5–10 mm^3^) to large cysts (91–150 mm^3^), with signal persistence extending to 48 hours post-injection, consistent with high-affinity binding and prolonged retention within collagen-rich fibrotic microenvironments. This contrasts sharply with the rapid washout and nonspecific signal characteristics of conventional GBCAs, which lack target engagement (Fig. 6 D–J).

Importantly, the strong quantitative correlation between hProCA32.Collagen MRI signal and histological collagen burden (CPA%; R^2^ = 0.55), compared with no meaningful correlation for non-targeted MRI (R^2^ ≈ 0.008), provides rigorous molecular validation of imaging specificity, meeting key criteria for imaging biomarker qualification, including biological specificity, quantitative accuracy, and reproducibility. These results position hProCA32.Collagen as a molecular imaging biomarker rather than just a surrogate anatomical readout.

Current state-of-the-art MRI approaches for fibrosis assessment, including diffusion-weighted imaging, BOLD MRI, magnetization transfer, arterial spin labeling, and MR elastography, provide indirect or composite measures of tissue structure, perfusion, and stiffness but lack molecular specificity for collagen deposition and are often confounded by inflammation, edema, or hemodynamic variability ^35, 36, 88, 89^. Similarly, TKV-based MRI metrics, which remain the clinical standard for ADPKD staging, capture macroscopic cyst burden but fail to detect microcysts and early fibrotic remodeling, resulting in delayed and indirect assessment of disease progression^3, 34, 90, 91^. By directly targeting collagen I, the dominant extracellular matrix component of fibrosis, hProCA32.Collagen overcomes these limitations, enabling spatially resolved, molecularly specific imaging of early fibrotic activity.

Extensive efforts have been devoted to developing collagen-targeted contrast agents by the addition of a molecular biomarker-targeting moiety to approved small chelator-based contrast agents^92–96^, but none have been approved due to challenges in trading off metal stability with relaxation properties, and limited expression of molecular biomarkers such as receptors and collagen (sub-nM-μM)^37, 68–76^. EP-3533, with conjugation of three DPTAs to the amide groups from a peptide targeting moiety for collagen, provided more information than non-specific Gd^3+^-based agents in a murine model of ischemia-reperfusion, with the ability to detect collagen-rich scar tissue 6 weeks after MI^97^, but translational applications have been limited due to tissue retention of Gd^3+^ from the linear chelator^98, 99^. Another agent, CM101, with 3 ProHance conjugated to a peptide to target collagen, was recently developed to detect increases in liver fibrosis after treatment, but its application in detecting renal fibrosis has not been reported^100–102^. Agents targeting other overexpressed extracellular matrix proteins, including elastin CNA35, have been reported^103, 104^. ESMA, an elastin-binding contrast agent, was used to visualize the renal fibrosis area^105^ similar to that measured with a conventional Gd^3+^-based contrast agent (GBCA)^106^. Waghorn et al. used an oxyamine-functionalized derivative of Gd^3+^-DOTA capable of undergoing reversible condensation reactions with aldehydes to target allysine in oxidized collagen^107, 108^. Caravan reported a promising allysine-binding Gd^3+^-oxyamine (OA) probe targeting oxidized collagen formed during fibrogenesis in CKD mouse models. However, these molecular targeted agents require high injection doses. Further, risks associated with the high dose of Gd^3+^ necessary to overcome low relaxivity, and applications to ADPKD with cysts have yet to be addressed.

A particularly novel contribution of this work is the noninvasive, region-resolved mapping of fibrosis–cyst interactions across renal compartments and the noninvasive uncovering and precision mapping of focal collagen deposition with quantitative MRI–histology concordance, enhanced microcyst detection and unmasking of renal fibrotic striation. Our data also demonstrate that cortical microfibrosis and outer stripe of the outer medulla (OSOM)-predominant cyst expansion represent spatially distinct but biologically interconnected processes, supporting emerging evidence that periglomerular and peritubular fibrosis precedes and actively contributes to cyst initiation and expansion. Mechanistically, early extracellular matrix stiffening may alter tubular biomechanics, promote epithelial proliferation, and facilitate tubular dilation, thereby linking fibrosis directly to cystogenesis rather than representing a secondary consequence. This level of spatial and mechanistic resolution is inaccessible with conventional imaging or volumetric metrics. This discovery is supported by detailed histopathological and segmented analyses of human ADPKD kidneys at various disease stages, combined with longitudinal studies in animal models, including orthologous rodent models (Pkd2 conditional knockout mice) and naturally occurring models (PCK rat, cpk mouse, jck mouse). Using segmented or endpoint approach, interstitial fibrosis is detectable very early in disease pathogenesis, often preceding or coinciding with the earliest detectable cysts ^61, 109^. Histopathological studies of human ADPKD kidneys have demonstrated that interstitial fibrosis and tubular atrophy (IFTA) are present even in kidneys with preserved overall function (eGFR >60 mL/min/1.73m^2^), indicating that structural damage precedes measurable GFR decline ^33, 110^. These findings suggest that fibrotic remodeling of the non-cystic interstitium, rather than simple mass effect from expanding cysts, may be the primary contributor of nephron loss and functional impairment in PKD ^6, 111^.

The relationship between cyst formation and fibrosis in ADPKD is increasingly recognized as bidirectional and temporally dynamic, challenging the traditional paradigm in which fibrosis is viewed solely as a late secondary response to cyst expansion ^112, 113^. Consistent with prior histopathological studies in human ADPKD and orthologous animal models, including Pkd1 and Pkd2 knockout mice and the PCK rat, interstitial fibrosis is detectable at early disease stages and often precedes or coincides with initial cyst formation. Our imaging findings provide the first in vivo, noninvasive confirmation of this paradigm, demonstrating that early fibrotic remodeling can be spatially mapped and quantitatively measured prior to detectable changes in organ function or volume.

Beyond renal disease, hProCA32.Collagen enables simultaneous staging of hepatic fibrosis, addressing a critical unmet need in ADPKD, where hepatic cystogenesis and biliary fibrosis contribute substantially to morbidity but remain poorly quantified noninvasively. The observed increase in hepatic CPA% (~30% vs ~10%, p < 0.001), together with sustained liver enhancement up to 48 hours, demonstrates that collagen-targeted pMRI provides a unified platform for multi-organ fibrosis assessment, which is not achievable with current imaging approaches.

The very important feature of our developed Gd-hProCA32.collagen contrast agent is its potential strong safety profile in kidney patient applications. Our injection dose is > 20-fold lower than approved Gadavist, which is approved for use in kidney patients with caution. hProCA32.collagen exhibits no cytotoxicity and tissue toxicity upon injection of hProCA32.collagen in rats, complete excretion from *in vivo*, strong metal-binding capability, and largely reduced injections for *in vivo* imaging. Taken together, hProCA32.collagen has strong potential to mitigate injury response and provide a sensitive and safe way for early detection, staging, and ADPKD clinical management and future therapeutic drug development. Future studies are required to validate our findings across orthologous PKD1 models and in human patients, overcoming challenges related to FDA IND approval. In addition, clinically relevant parameters remain to be properly assessed, including pharmacokinetics, dosing optimization, and safety in clinical settings.

In conclusion, this work establishes collagen-targeted precision MRI as a transformative approach for early detection and staging of fibrosis in ADPKD, enabling direct visualization of a previously inaccessible subclinical disease phase. By bridging the gap between molecular pathology and noninvasive imaging, hProCA32.Collagen has the potential to redefine clinical trial endpoints, improve patient stratification, and accelerate the development of antifibrotic therapies across CKD and other fibrotic diseases.

## Methods and Methods

### Animal Models and Small Animal MRI

All procedures using rats and mice were approved by the Institutional Animal Care and Use Committee (IACUC) at the University of Alabama at Birmingham (approval #20096 and #21072, respectively). To model the renal and hepatic manifestations of ADPKD, we utilized the Pkhd1^*pck/pck*^ (PCK) rat model, which harbors a spontaneous mutation in the *Pkhd1* gene orthologous to human ADPKD and recapitulates the progressive cystic expansion, interstitial fibrosis, and renal functional decline characteristic of human polycystic kidney disease ^86, 114^Age-matched Sprague-Dawley (SD) rats obtained from Charles River Laboratories (Wilmington, MA, USA) served as wild-type controls. A total of 25 male rats were studied, comprising 15 PCK rats and 10 SD control rats, all within the age range of 11– 41 weeks to ensure comparable disease stage and minimize age-related confounding variables.

*Pkd2*^fl/fl^ mice (Jackson Laboratory Stock #017292) were crossed with CaggCre^ER^ mice to establish *Pkd2*^fl/fl^; CaggCre^ER^ conditional mutants. To delete *Pkd2*, mice were injected intraperitoneally with three daily injections of tamoxifen (6mg/40g body weight, Sigma-AldrichCat# T5648–5G) starting at 8 weeks of age. Tamoxifen was dissolved in corn oil, and the mixture was rocked at 37°C until it was dissolved. All mutants and control mice were injected with tamoxifen. A total of 4 mice were studied, comprising 2 Cre^+^ rats and 2 Cre^−^ mice.

All animal imaging was performed at the Small Animal Imaging Core Facility (SAIF) at the University of Alabama at Birmingham (UAB). Animals were anesthetized with 1.5–2% isoflurane, with respiratory rates maintained at 47–50 breaths per minute throughout all imaging sessions. Animals were positioned in the prone position, headfirst, on a temperature-regulating bed to maintain physiological body temperature and imaged in the axial plane using a 9.4 T small animal MRI scanner (Bruker BioSpec, Billerica, MA) equipped with an 86 mm volume coil. Multiparametric MRI data were acquired *in vivo* using the following optimized pulse sequence protocol. T2-weighted anatomical imaging was performed using a 2D RARE sequence (TR = 3313 ms; TE = 21.25 ms; flip angle = 90°; FOV = 64 × 64 mm; matrix = 320 × 128; RARE factor = 4; slice thickness = 1 mm; number of averages = 2). T1-weighted anatomical imaging was acquired using a 2D RARE sequence (TR = 1425 ms; TE = 12 ms; flip angle = 90°; FOV = 64 × 64 mm; matrix = 320 × 128; RARE factor = 4; slice thickness = 1 mm; number of averages = 2). Three-dimensional T2-weighted RARE imaging was performed for accurate volumetric cyst measurement (TR = 1000 ms; TE = 80 ms; FOV = 128 × 64 × 64 mm; matrix = 320 × 128 × 64; RARE factor = 8). T1 mapping was performed using a FAIR RARE inversion recovery sequence (TR = 5000 ms; TE = 45.18 ms; FOV = 64 × 64 mm; matrix = 320 × 128; RARE factor = 16; slice thickness = 1 mm; number of averages = 2), with multiple inversion times (TI = 50, 300, 600, 1000, 1500, 1800, 2000, 3000). T2 mapping was acquired using a 2D multi-slice multi-echo (MSME) sequence (TR = 5009 ms; TE = 9.2 ms; FOV = 64 × 64 mm; matrix = 320 × 128; slice thickness = 1 mm; number of averages = 2).

For contrast-enhanced imaging, hProCA32.Collagen was administered intravenously via tail vein injection at a dose of 0.010 mmol/kg at an injection rate of 1 mL/min to PCK rats (n = 8) and SD control rats (n = 3). Gadovist (gadobutrol; Bayer Healthcare, Wayne, NJ) was administered at a standard clinical dose of 0.10 mmol/kg via the same route to a separate cohort of PCK rats (n = 5) and SD control rats (n = 3) for direct comparison. Longitudinal MRI data were acquired at pre-injection baseline and at 0, 3, 24, and 48 hours post-injection to characterize contrast agent pharmacokinetics and tissue retention profiles across the full time course.

For MRI, mice were positioned prone in a Bruker BioSpec 9.4T MRI scanner, and multiparametric MRI sequences (T1W_RARE and T2W_RARE) were acquired at pre-injection baseline. Gd-hProCA32.Collagen was administered intravenously via tail vein injection at a dose of 0.010 mmol/kg at an injection rate of 1 mL/min, and longitudinal MRI data were acquired at pre-injection baseline and at 1 and 24 hours post-injection to characterize contrast agent pharmacokinetics and tissue retention profiles.

At the terminal timepoint, kidneys and livers were isolated, fixed in 10% neutral buffered formalin, embedded in paraffin, and sectioned for hematoxylin and eosin (H&E) and Sirius Red staining using standard procedures.

All experimental procedures were performed in strict accordance with the National Institutes of Health Guide for the Care and Use of Laboratory Animals. All protocols were reviewed and approved by the Institutional Animal Care and Use Committee (IACUC) of the University of Alabama at Birmingham (UAB). The University of Alabama at Birmingham is fully accredited by the Association for Assessment and Accreditation of Laboratory Animal Care International (AAALAC).

### *Ex vivo* MRI

Following completion of longitudinal *in vivo* imaging, animals were euthanized, and the kidneys were immediately harvested and prepared for *ex vivo* MRI analysis. Excised kidneys were immersed in phosphate-buffered saline (PBS) to maintain tissue hydration and structural integrity during imaging. *Ex vivo* imaging was performed on a 9.4 T small animal MRI scanner (Bruker BioSpec, Billerica, MA) equipped with a 38 mm volume coil at the Small Animal Imaging Core Facility (SAIF), University of Alabama at Birmingham.

*Ex vivo* T1-weighted imaging was acquired using a 2D RARE sequence (TR = 938 ms; TE = 12 ms; flip angle = 180°; FOV = 50 × 8 mm; matrix = 320 × 128; RARE factor = 2; slice thickness = 1 mm; number of averages = 2). *Ex vivo* T2-weighted imaging was acquired using a 2D RARE sequence with identical parameters (TR = 3000 ms; TE = 21.25 ms; flip angle = 180°; FOV = 50 × 8 mm; matrix = 320 × 128; RARE factor = 4; slice thickness = 1 mm; number of averages = 2). Phantom T1-weighted imaging was acquired using 2D RARE sequence (TR = 512.82 ms; TE = 12 ms; flip angle = 180°; FOV = 63 × 10 mm; matrix = 320 × 128; RARE factor = 2; slice thickness = 0.5 mm; number of averages = 2). T2-weighted imaging was acquired using a 2D RARE sequence with identical parameters (TR = 3000 ms; TE = 21.25 ms; flip angle = 180°; FOV = 63× 10 mm; matrix = 320 × 128; RARE factor = 4; slice thickness = 0.5 mm; number of averages = 2).

### Conventional Data Acquisition for PKD

Body weight (g) was recorded for each animal following every MRI imaging session to monitor physiological status throughout the longitudinal study. Upon study completion, animals were euthanized, and the kidneys and liver were immediately harvested and weighed (g) to assess organ-level disease burden and correlate with MRI-derived volumetric measurements.

### MRI Data Analysis

All signal-to-noise ratio (SNR) and contrast-to-noise ratio (CNR) analyses were performed using ImageJ software (version 1.53, National Institutes of Health, Bethesda, MD; freely available at imagej.nih.gov). For each imaging timepoint, regions of interest (ROIs) were manually delineated by an experienced observer in the renal cortical parenchyma to quantify signal intensity, while a reference ROI was placed in a region of inhomogeneous background air outside the animal to estimate image noise as the standard deviation of background signal. ROIs were systematically drawn across all image slices, capturing the full kidney anatomy, and were carefully matched to the anatomical appearance of the pre-injection (0 h) baseline timepoint for each animal to ensure consistent spatial sampling throughout the longitudinal study. To minimize confounding contributions from non-parenchymal structures, major renal blood vessels, the inner medullary region, and the papilla were carefully excluded from all ROI selections. For longitudinal comparison across timepoints, post-injection images were visually co-registered side-by-side with their corresponding pre-injection baseline images to identify slices with consistent anatomical landmarks, ensuring that signal changes reflected true contrast enhancement rather than slice position variability.

The signal-to-noise ratio (SNR) was calculated using the formula, assuming magnitude-reconstructed images:

SNR=(SignalintensityofROI)/(Standarddeviationofbackground)

The MRI percent signal enhancement over time was calculated as:

$$\%\;\mathrm{Enhancement}\;=\left(\frac{SNR\;\mathrm{post}\;-\;\mathrm{SNR}\;\mathrm{pre}\;}{\;\mathrm{SNR}\;\mathrm{pre}\;}\right)\times 100$$

Contrast-to-noise ratio (CNR) was computed using:

$$CNR=\frac{\left(\mathrm{Signal}\;\mathrm{intensity}\;\mathrm{of}\;\mathrm{ROI}\;-\;\mathrm{Signal}\;\mathrm{Intensity}\;\mathrm{of}\;\mathrm{reference}\right)}{\mathrm{Standard}\;\mathrm{deviation}\;\mathrm{of}\;\mathrm{background}}$$

where the reference was either a muscle ROI or a tissue-matched baseline ROI.

Area under the curve (AUC) measurements were performed to quantify the total longitudinal MRI signal enhancement for each contrast agent across all post-injection timepoints. AUC calculations were conducted in R statistical software (version 4.3.1, R Foundation for Statistical Computing, Vienna, Austria; available at r-project.org) using the pracma::trapz() function, which implements the trapezoidal numerical integration method to compute the cumulative area under the SNR enhancement curve as a function of time.

### Organ Weight and Total Kidney Volume Assessment

At the experimental endpoint, PCK and WT rats (n = 7 per group) were weighed and sacrificed under isoflurane anesthesia. Kidneys and liver were surgically excised, blotted dry, and weighed on a precision analytical balance. Kidney weight-to-body weight (KW/BW) and total kidney volume-to-body weight (TKV/BW) were calculated for each animal. Total Kidney Volume (TKV) was measured (comprising the volumes of both the left and right kidneys) using 3D Slicer and Imalytics Software. Manual Method (MM), which involves manually outlining the kidneys at T2W MRI images in the axial, sagittal, and coronal views, was used to outline the edge of the kidney in each image slice; a binary mask of the kidney was generated, and the TKV in mm^3^ was calculated by summing all voxels within the ROI. The TKV was also measured using the Ellipsoid formula method (EM) and compared with the MM as described ^114^.

Volume measurements of cysts were performed using 3D Slicer. The volume of the cysts in the cortex and the OSOM was measured by manually selecting the volume of interest (cyst area) and setting low/high signal intensity thresholds to highlight only the cysts. Using voxel dimensions derived from imaging resolution, the number of pixels was counted and converted to cyst volume. All kidney and cyst volume measurements were repeated twice for accuracy.

To capture the severity and account for the variability of the cystic kidney disease in the left and right kidneys, we analyzed the renal cystic burden for each rat. Cysts were categorized by volume as microcysts (0–10 mm^3^), small (11–30 mm^3^), medium (31–90 mm^3^), and large (91–150 mm^3^).

Agreement between T1-weighted and T2-weighted TKV measurements was assessed by Pearson correlation analysis. Statistical comparisons between PCK and WT groups were performed using unpaired Studenťs t-test, with p < 0.05 considered statistically significant.


$$\;\mathrm{Renal}\;\mathrm{cystic}\;\mathrm{burden}\;(RCB,\%)=\frac{\left(\mathrm{Total}\;\mathrm{cyst}\;\mathrm{volume}\;(\mathrm{TCV})\mathrm{right}\;+\;\mathrm{left}\;\mathrm{kidney}\right)}{\left(\mathrm{Total}\;\mathrm{Kidney}\;\mathrm{volume}\;\mathrm{(TKV)right}\;+\;\mathrm{left}\;\mathrm{kidneys}\right)}\times 100$$

To represent the proportion of the kidney volume occupied by the cyst, the fractional cyst volume (FCV) was calculated by dividing the total cyst volume (TCV) by the total kidney volume (TKV).


FractionalCystVolume(FCV)=(Totalcystvolumes(TCV))/(TotalKidneyvolumes(TKV))X100

The Cortex+OSOM was manually selected from the *ex vivo* MRI data using ImageJ, and the Cyst Index (CI) was analyzed as described ^115^.


$$\mathrm{Cyst}\;\mathrm{Index}\;(CI)=\frac{\mathrm{Total}\;\mathrm{Cyst}\;\mathrm{Area}}{\mathrm{Total}\;\mathrm{Area}}$$

The severity of fibrosis was analyzed from the Picrosirius red data to determine the fibrosis score as described ^116^.


$$\;\mathrm{Fibrosis}\;\mathrm{Score}\;=\frac{\mathrm{Total}\;\mathrm{Fibrosis}\;\mathrm{Area}}{\mathrm{Total}\;\mathrm{Area}}$$

The ratios of Kidney Weight (KW) to Body Weight (BW) and Kidney Size (KS) to Body Weight (BW) were analyzed as described^115^.


$$\frac{KW}{BW}\;=\frac{\mathrm{Weight}\;\mathrm{of}\;\mathrm{Kidney}}{\mathrm{Weight}\;\mathrm{of}\;\mathrm{Body}}\;\frac{KS}{BW}\;=\frac{\mathrm{Kidney}\;\mathrm{Size}}{\mathrm{Weight}\;\mathrm{of}\;\mathrm{body}}$$

### Serum Clinical Chemistry Analysis

Blood samples were collected from PCK rats at 11, 26, 34, and 41weeks of age and corresponding age-matched wild-type Sprague-Dawley controls (n = 7 per group) via cardiac puncture under isoflurane anesthesia. Serum was isolated by refrigerated centrifugation at 10,000 × g for 10 minutes at 4°C and stored at −20°C until analysis. Comprehensive serum biochemical profiling was performed by IDEXX BioAnalytics (North Grafton, MA, USA) using a validated veterinary clinical chemistry platform, encompassing renal function markers (creatinine, blood urea nitrogen (BUN), BUN/creatinine ratio, phosphorus), hepatic function markers (ALT, AST, GGT, ALP, albumin, globulin, total protein, total and unconjugated bilirubin, ALB/GLOB ratio), metabolic markers (sodium, potassium, chloride, calcium, bicarbonate TCO_2_, cholesterol, triglycerides, glucose), and enzymatic markers (lipase, amylase, LDH, and creatine kinase).

At each age group, serum biochemical parameters were independently compared between PCK rats and their corresponding age-matched WT Sprague-Dawley controls using unpaired two-tailed Studenťs t-tests. Since each age group comparison (PCK 11 weeks vs. WT 11 weeks; PCK 26 weeks vs. WT 26 weeks; PCK 34 weeks vs. WT 26 weeks; PCK 41 weeks vs. WT 26 weeks) represents an independent between-group analysis, one-way ANOVA with Tukey’s post-hoc correction was applied when comparing across all three age groups simultaneously to account for multiple comparisons. A p-value of < 0.05 was considered statistically significant for all analyses. All statistical analyses were performed using GraphPad Prism (version 10.0, GraphPad Software, San Diego, CA, USA).

### Histological Analysis and Fibrosis Quantification

For histological analysis, harvested organs were fixed overnight in 10% neutral buffered formalin, followed by transfer into 70% ethanol to ensure optimal tissue preservation and morphological integrity. Fixed tissues were subsequently processed through standard dehydration and clearing protocols, paraffin-embedded, and sectioned in the axial plane at 5 μm thickness using a calibrated rotary microtome. Serial sections were mounted on positively charged glass slides and stained using the following validated histological protocols: Hematoxylin and Eosin (H&E) staining for assessment of overall tissue architecture, cellular morphology, cyst distribution, and inflammatory infiltration; and Picrosirius Red staining for quantitative assessment of fibrillar collagen deposition (collagen I and III), performed according to the manufacturer’s protocol (Abcam, Cambridge, UK). Stained sections were imaged using a brightfield microscope, and CPA% was quantified using ImageJ software (National Institutes of Health, Bethesda, MD) by thresholding Picrosirius Red-positive pixels relative to total tissue area across a minimum of five representative fields per section. All statistical analyses were performed using GraphPad Prism (version 11).

### Statistical Analysis

All statistical analyses were performed using R (version 4.3.1, R Foundation for Statistical Computing, Vienna, Austria) and GraphPad Prism (version 11.0.2, GraphPad Software, San Diego, CA). Continuous variables are reported as mean ± standard deviation (SD) or mean ± standard error of the mean (SEM) as indicated in figure legends. All tests were two-sided, and p < 0.05 was considered statistically significant.^117, 118^

For longitudinal MRI measurements, including signal-to-noise ratio (SNR), contrast-to-noise ratio (CNR), and percent signal enhancement, data were analyzed using linear mixed-effects models to account for repeated measurements within the same animal. Fixed effects included group (PCK versus WT), imaging timepoint (pre-injection, 3, 24, and 48 hours post-injection), and their interaction (group × timepoint). A random intercept was included per animal to model within-subject correlation. Model assumptions, including normality and homoscedasticity of residuals, were verified using standard diagnostic plots. Post hoc pairwise comparisons were performed using estimated marginal means with Tukey’s adjustment for multiple comparisons. Between-group comparisons at individual time points were performed using an unpaired Studenťs t-test or a Mann-Whitney U test, selected based on normality assessment using the Shapiro-Wilk test.

Receiver operating characteristic (ROC) curve analysis was performed in GraphPad Prism to assess diagnostic accuracy of Gd-hProCA32.Collagen versus Gadovist for discrimination of PCK fibrotic from WT kidneys using T2W MRI SNR enhancement as the classification variable. SNR values were extracted at the peak enhancement timepoint for each agent, 24 hours post-injection for Gd-hProCA32.Collagen, reflecting sustained collagen-targeted tissue retention, and 1 hour post-injection for Gadovist, reflecting its early peak enhancement before renal clearance, ensuring each agent was evaluated under optimal imaging conditions. AUC with 95% confidence intervals was calculated and tested against the null hypothesis of AUC = 0.5. The optimal diagnostic threshold was determined by Youden’s J index. Histological fibrosis was quantified as collagen proportionate area (CPA%) from Sirius Red-stained sections using ImageJ/Fiji morphometric analysis, and MRI-histology correlation was assessed by Pearson linear regression.

## Supplementary Material

Supplement 1

Figs. S1 to S12

Table S1

## Data Availability

All data and code needed to evaluate and reproduce the conclusions in the paper are present in the paper and/or the Supplementary Materials, and additional data related to this paper may be requested from the corresponding authors. No new materials were generated in this study.
