# Supplementary material for: Unmasking Early Renal Fibrosis in Polycystic Kidney Disease Using Noninvasive Precision Molecular MRI of Collagen
Source: bioRxiv. 2026 Jul 20:2026.07.19.739445. Preprint. [Version 1] doi: 10.64898/2026.07.19.739445 (PMC13419732; doi:10.64898/2026.07.19.739445)
Supplement: Supplementary file 1 [file media-1.pdf]

Supplemental materials for

Unmasking Early Renal Fibrosis in Polycystic Kidney Disease Using Noninvasive Precision Molecular MRI of Collagen

Oluwabukola Bamishaye, et al.

Corresponding authors:

Jenny J. Yang; jjyang2@uab.edu

Michal Mrug: mmrug@uabmc.edu

The PDF file includes:

Figs. S1 to S12

Table S1

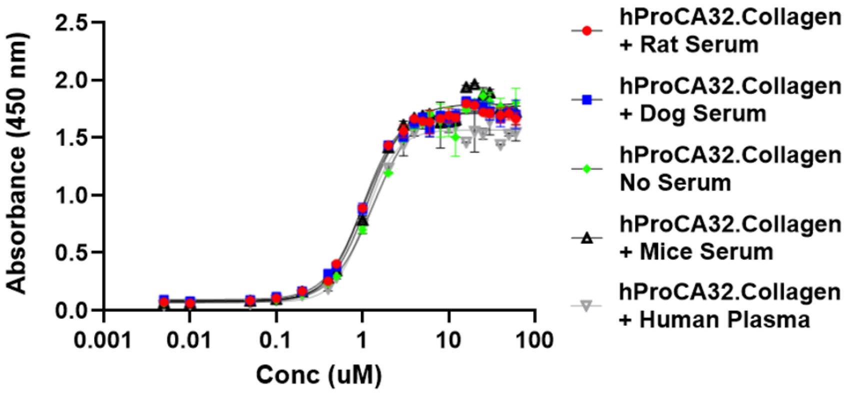

Best-fit values	hProCA32.Collagen + Rat Serum	hProCA32.Collagen + Dog Serum	hProCA32.Collagen No Serum	hProCA32.Collagen + Mice Serum	hProCA32.Collagen + Human Plasma
Bottom	0.080	0.091	0.068	0.078	0.07180
Hillslope	2.127	1.978	1.817	1.991	2.442
Top	1.712	1.724	1.762	1.791	1.566
EC50 (μM)	**0.998**	**1.029**	**1.336**	**1.145**	**1.092**
logEC50	-0.001	0.012	0.126	0.059	0.038
Span	1.631	1.634	1.694	1.712	1.494

Figure S1. Collagen-binding affinity of hProCA32.Collagen is preserved across serum conditions. Enzyme-linked immunosorbent assay (ELISA) binding curves of hProCA32.Collagen to collagen type I across a concentration range of 0.001–100 μM, measured in the presence of rat serum, dog serum, mouse serum, human plasma, or no serum (n = 3 per condition; mean ± SEM). All conditions produced superimposable sigmoidal dose-response curves, with EC_{50} values ranging narrowly from 0.998 to 1.336 μM (rat serum: 0.998 μM; dog serum: 1.029 μM; no serum: 1.336 μM; mouse serum: 1.145 μM; human plasma: 1.092 μM), as determined by four-parameter nonlinear regression (best-fit parameters shown in the table). Hill slopes near 2 indicate cooperative binding behavior. The negligible shift in EC_{50} across all serum and plasma conditions demonstrates that endogenous serum proteins do not competitively inhibit collagen binding, supporting the translational applicability of hProCA32.Collagen across preclinical species and into the human setting.

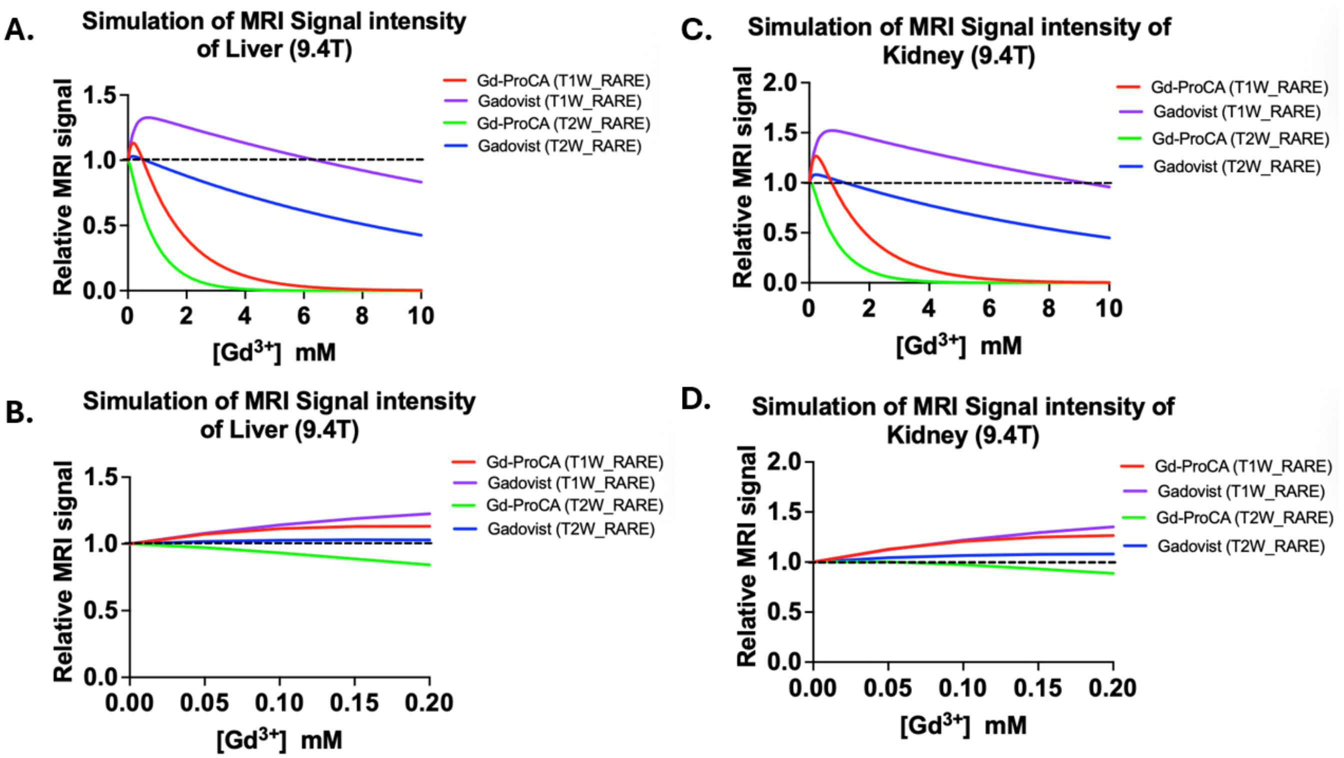

Figure S2. Simulated MRI signal intensity as a function of gadolinium concentration for Gd-ProCA and Gadovist in the liver and kidney at 9.4T. Theoretical MRI signal intensity (relative to pre-contrast baseline, dashed line) was modeled as a function of Gd^{3+} concentration (0–10 mM) for the collagen-targeted agent Gd-ProCA and the clinical extracellular agent Gadovist, across T1-weighted (T1W_RARE) and T2-weighted (T2W_RARE) acquisition sequences at 9.4T. Simulations were performed for (A, B) liver and (C, D) kidney tissue. Panels (A) and (C) display the full concentration range (0–10 mM), revealing divergent signal behavior: T1W sequences produce a transient signal enhancement at low Gd^{3+} concentrations, followed by signal loss at higher concentrations, whereas T2W sequences cause progressive signal attenuation across all concentrations for both agents. Panels (B) and (D) expand the physiologically relevant low-concentration range (0–0.20 mM). These simulations inform optimal sequence selection for detecting collagen-targeted contrast enhancement *in vivo* at high field strength.

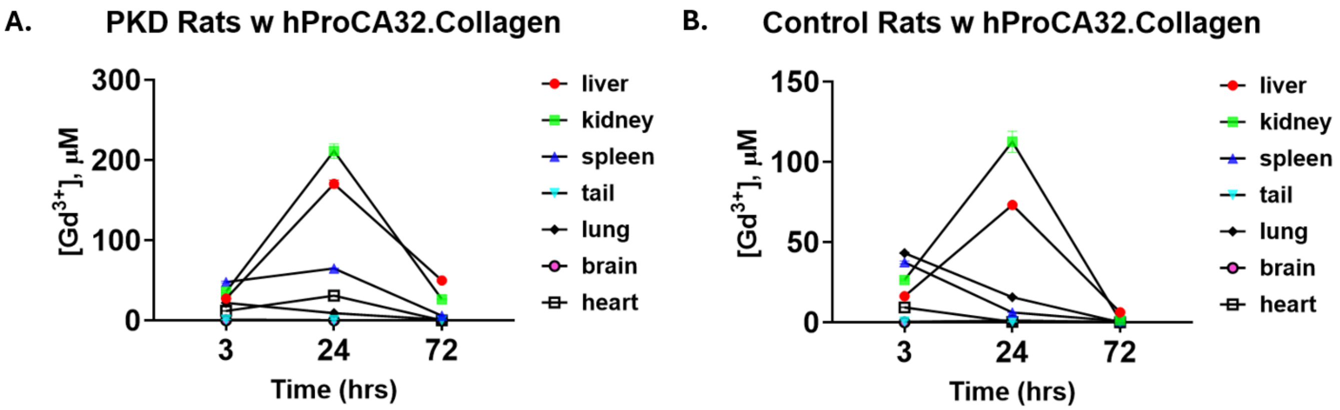

Figure S3. Biodistribution and clearance kinetics of hProCA32.Collagen in PCK and control rats. (A) Time-dependent gadolinium concentration ([Gd^{3+}], μM) in major organs of PCK rats following administration of hProCA32.Collagen. Peak accumulation was observed at 24 h post-injection in the kidneys and liver, with lower uptake in the spleen, lung, heart, tail, and brain. Substantial clearance was observed by 72 h. (B) Organ biodistribution profile in control rats demonstrating lower overall retention compared with PKD rats, particularly in the kidneys and liver. Minimal brain accumulation was observed in both groups, indicating limited blood–brain barrier penetration. Data demonstrate preferential accumulation and prolonged retention of hProCA32.Collagen in fibrotic ADPKD-associated organs with subsequent systemic clearance over time.

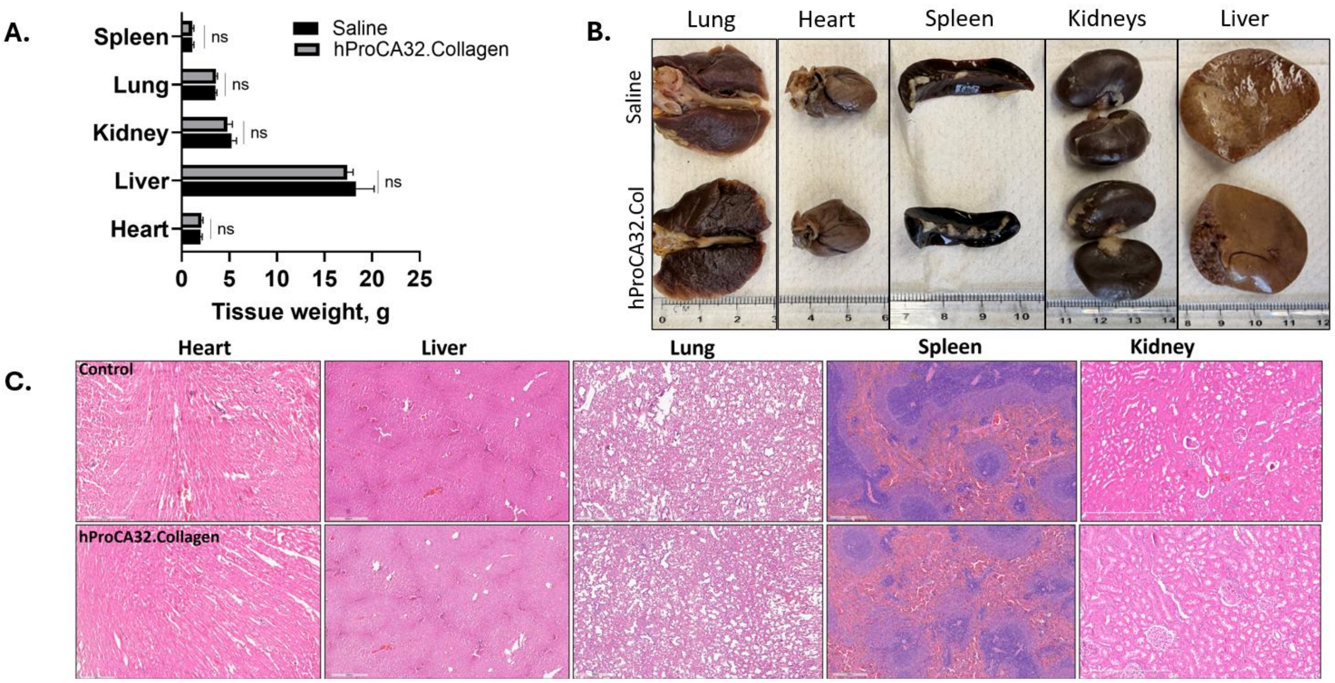

Figure S4. *In vivo* toxicological assessment of hProCA32.Collagen in Rats. (A) Organ weight comparison between saline-treated control rats and hProCA32.Collagen-treated rats across five major organs (heart, liver, kidney, lung, and spleen), harvested 4 days post-injection. No statistically significant differences in tissue weight were observed between groups (ns; $p > 0.05$), indicating the absence of organ-level toxicity following hProCA32.Collagen administration. Data are presented as mean ± SEM. (B) Representative gross morphological photographs of harvested organs collected 4 days post-injection (lung, heart, spleen, kidneys, and liver) from saline-treated (top row) and hProCA32.Collagen-treated (bottom row) rats. No gross morphological abnormalities, discoloration, or structural changes were observed in any organ following contrast agent administration. Scale bars shown. (C) Representative hematoxylin and eosin (H&E)-stained histological sections of heart, liver, lung, spleen, and kidney collected 4 days post-injection from control (top row) and hProCA32.Collagen-treated (bottom row) rats. No histopathological abnormalities, inflammatory infiltrates, necrosis, or tissue damage were detected in any organ examined, confirming the biocompatibility and safety profile of hProCA32.Collagen at the imaging dose administered.

Table S1. Serum chemistry analysis in wild-type and PCK rats. Comprehensive serum biochemistry profiles were obtained from wild-type (WT) and polycystic kidney disease (PCK) rats at progressive disease stages (11, 26, 34, and 41 weeks) to evaluate systemic toxicity, hepatic function, renal function, electrolyte balance, and metabolic status following hProCA32.Collagen administration. Measured parameters included liver enzymes (ALT, AST, ALP, GGT), renal biomarkers (creatinine, BUN), pancreatic enzymes (amylase, lipase), electrolytes, proteins, lipids, and metabolic markers. Reference ranges for each parameter were obtained from Charles River Laboratories normative databases for age- and strain-matched Sprague-Dawley rats and are provided alongside measured mean values for direct comparison.

	WT Rat	PCK 11 Weeks	PCK 26 Weeks	PCK 34 Weeks	PCK 41 Weeks	REFERENCE (RANGE) MEAN
**Creatine Kinase (U/L)**	1328	1131	1009	780.5	211.0	(%6–477) 241
**Amylase (U/L)**	662.0	847.0	781.7	821.0	-	(500–1200) 850
**Glucose (mg/dL)**	414.3	123.5	538.0	290.0	158.5	(92–138) 115
**LDH (U/L)**	444.3	366.0	364.7	249.0	-	(76–953) 210
**GGT (U/L)**	0.000	0.333	1.000	1.000	-	(0–3) 0.5
**Albumin (g/dL)**	3.400	2.950	2.533	2.450	2.150	(3.7–5.0) 4.45
**Total Bilirubin (mg/dL)**	0.100	0.100	0.100	0.150	0.700	(0.10–0.20) 0.15
**Total Protein (g/dL)**	7.333	5.200	6.333	6.400	6.950	(6.3–8.1) 6.7
**Globulin (g/dL)**	3.933	2.800	3.800	3.950	4.800	(1.5–3.0) 2.2
**Creatinine (mg/dL)**	0.300	0.373	0.713	0.585	0.230	(0.4–0.6) 0.57
**Phosphorus (mg/dL)**	9.900	8.950	8.733	7.200	4.900	(5.50–9.20) 7.65
**Potassium (mmol/L)**	8.433	8.333	8.633	8.050	4.800	(4.2–6.7) 5.65
**ALB/GLOB Ratio**	0.867	0.100	0.667	0.600	0.500	(0.8–2.0) 1.2
**Bilirubin (mg/dL)**	0.100	0.100	0.100	0.100	0.600	(0.0–0.15) 0.08
**Uric Acid (mg/dL)**	8.533	8.333	9.033	7.250	12.00	(1.2–7.5) 3.5
**ALP (U/L)**	131.0	141.5	171.0	150.0	239.5	(53–226) 105
**AST (U/L)**	120.7	86.00	118.3	83.50	169.0	(57–144) 90.5
**ALT (U/L)**	62.67	37.50	58.33	79.50	80.00	(26–97) 47
**Lipase (U/L)**	31.33	64.33	77.00	82.50	-	(20–160) 60
**BUN (mg/dL)**	18.67	16.00	23.67	35.00	16.00	(15–80) 40
**Cholesterol (mg/dL)**	97.33	100.0	132.3	157.5	73.50	(62–234) 92
**Calcium (mg/dL)**	12.90	8.567	12.13	12.45	9.900	(9.9–11.9) 10.9
**Bicarbonate TCO2 (mmol/L)**	26.00	22.00	24.33	25.00	31.00	(18–28) 23
**Chloride (mmol/L)**	94.67	92.00	89.67	100.5	100.5	(95–111) 103.5
**Sodium (mmol/L)**	143.7	144.0	138.0	144.5	140.5	(143–157) 148
**BUN/Creatinine Ratio**	62.97	48.40	37.87	60.00	70.20	(15–80) 40
**NA/K Ratio**	17.00	14.33	12.33	18.00	29.00	(15–30) 22
**Triglycerides (mg/dL)**	226.7	217.7	197.3	236.5	-	(46–208) 94

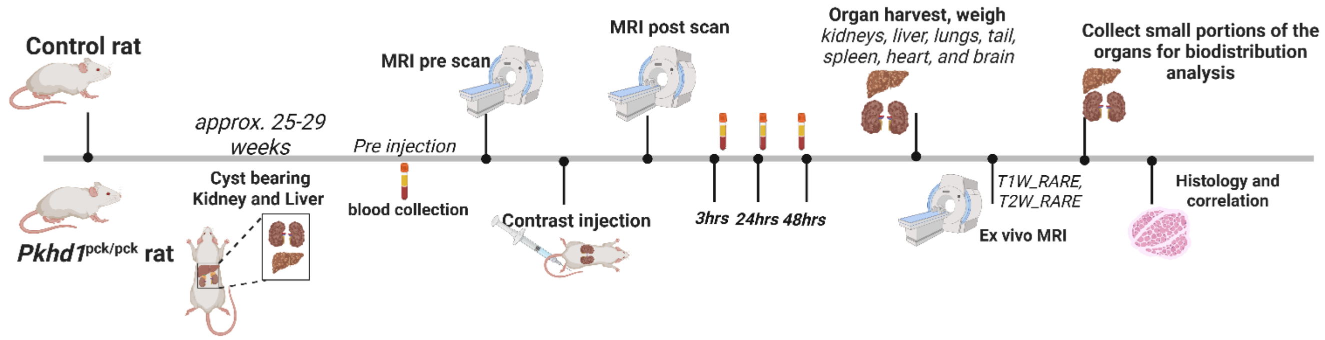

Figure S5. Experimental design and imaging workflow for *in vivo* evaluation of Gd-hProCA32.Collagen vs Gadovist in the *Pkhd1*$^{pck/pck}$ rat model of ADPKD. Schematic timeline depicting the study design for wild-type (WT) control rats and cyst-bearing *Pkhd1*$^{pck/pck}$ (PCK) rats at approximately 25–29 weeks of age, at which stage rats exhibit established renal and hepatic cystic burden. Blood was collected at pre-injection baseline, followed by multiparametric MRI pre-scan acquisition including T1W_RARE, T2W_RARE, T1M, and T2M sequences. Gd-hProCA32.Collagen was administered by intravenous injection, and post-contrast MRI (T1W_RARE, T2W_RARE, T1M, T2M) was acquired at 3, 24, and 48 hours post-injection. Following the final *in vivo* imaging session, kidneys, liver, lungs, tail, spleen, heart, and brain were harvested and weighed. Small tissue portions were collected from each organ for biodistribution analysis. *Ex vivo* MRI (T1W_RARE, T2W_RARE) was subsequently performed on excised kidneys and liver to enable high-resolution structural and contrast enhancement mapping without respiratory motion. Histological sections were obtained from all harvested organs for Sirius Red, Masson's Trichrome, and H&E staining, with MRI-histology spatial correlation performed to validate collagen-targeted signal enhancement. *Created in BioRender. Yang lab, J. (2026) https://BioRender.com/z4ey04m*

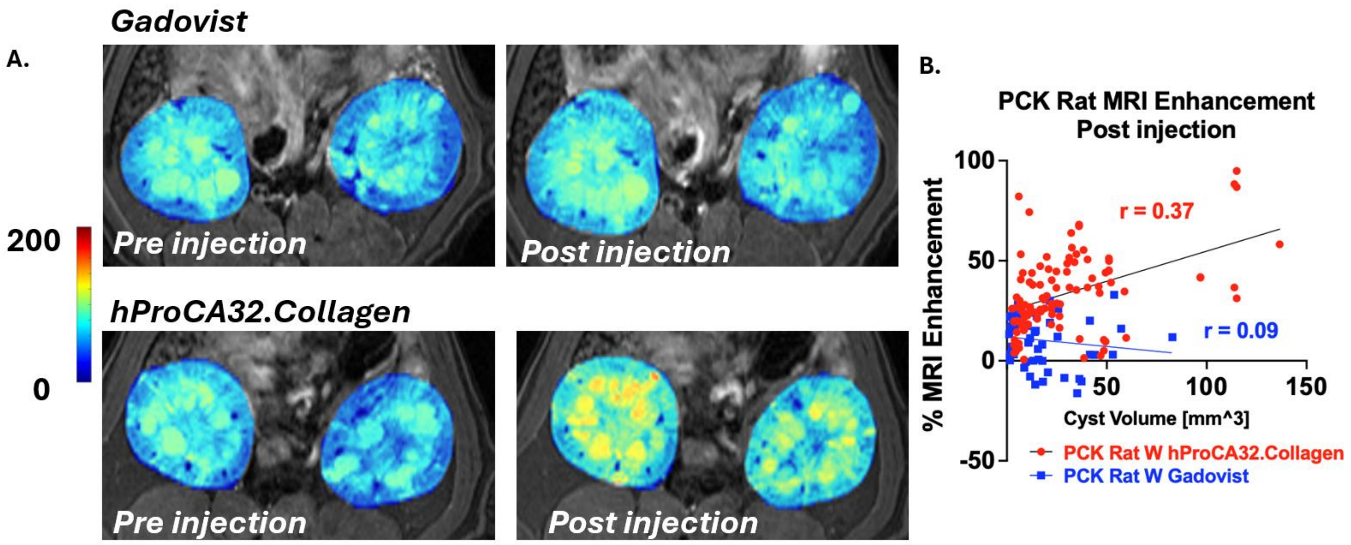

Figure S6. hProCA32.Collagen produces sustained T2-weighted MRI enhancement in PCK rat kidney cysts with signal correlated to cyst volume. (A) Representative T2-weighted MRI images of PCK rat kidneys at pre-injection, 24 hours, and 48 hours post-injection for hProCA32.Collagen (bottom row, 0.010 mmol/kg) and at pre-injection, 1 hour, and 24 hours post-injection for Gadovist (top row, 0.10 mmol/kg). hProCA32.Collagen produced progressive and sustained intracystic signal enhancement that persisted for 48 hours, whereas Gadovist showed only transient signal change that dissipated by 24 hours, consistent with rapid clearance in the absence of collagen-specific retention. (B) Percent MRI enhancement versus cyst volume (mm^3) in PCK rats following hProCA32.Collagen (red circles; $r = 0.37$) or Gadovist (blue squares; $r = 0.09$) administration. The positive correlation for hProCA32.Collagen, absent with Gadovist, confirms volume-dependent collagen-targeted molecular binding rather than nonspecific extracellular distribution. n = 8 PCK rats (hProCA32.Collagen); n = 5 PCK rats (Gadovist).

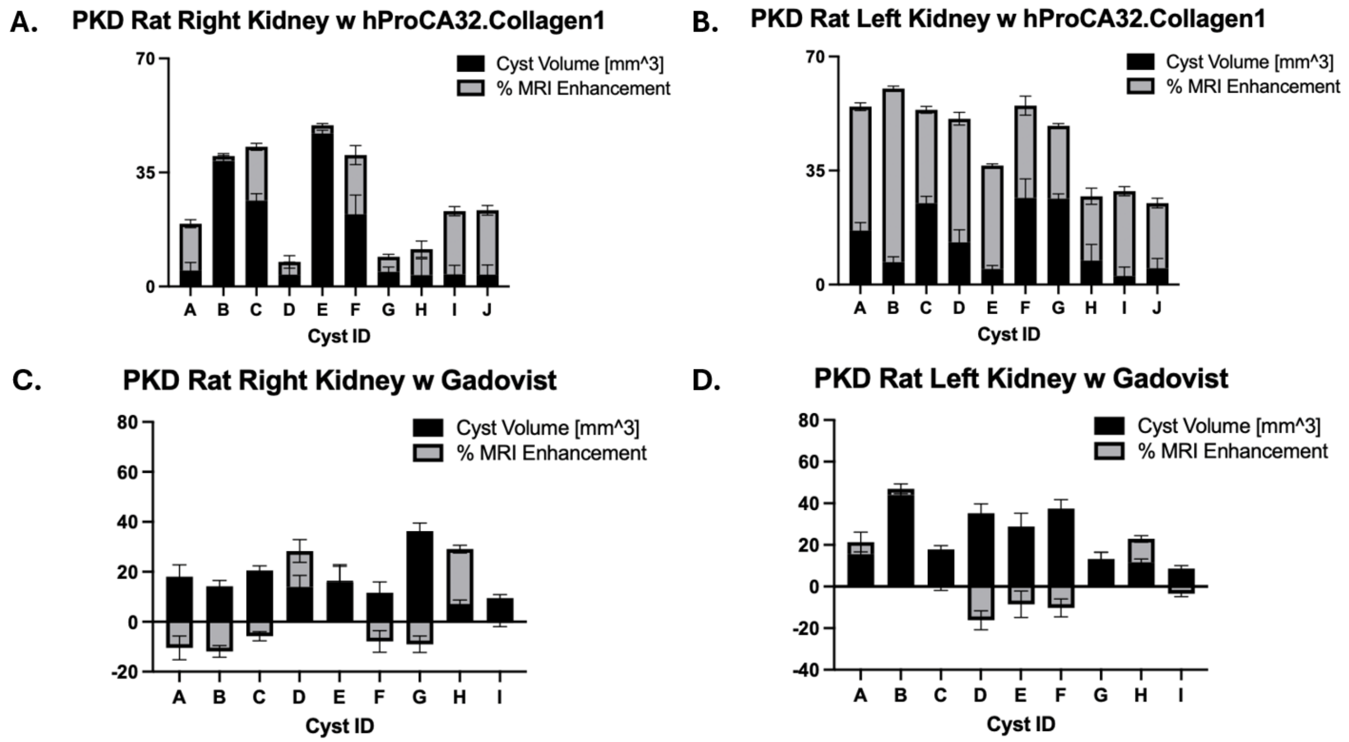

Figure S7. Individual cyst MRI enhancement profiles in PCK rat kidneys following hProCA32.Collagen or Gadovist administration. (A and B) Cyst volume (mm³, black bars) and percent MRI enhancement (%, gray bars) for individually tracked cysts (labeled A–J) in the right (A) and left (B) kidneys of PCK rats following hProCA32.Collagen administration (0.010 mmol/kg). Across both kidneys, hProCA32.Collagen produced consistently positive and substantial percent MRI enhancement across all individually resolved cysts, regardless of cyst volume, demonstrating uniform collagen-targeted signal amplification across the full cyst size spectrum. (C and D) Corresponding cyst volume and percent MRI enhancement profiles for individually tracked cysts (labeled A–I) in the right (C) and left (D) kidneys of PCK rats following Gadovist administration (0.10 mmol/kg). Data points represent mean ± SEM. n = 5 PCK rats (Gadovist); n = 8 PCK rats (hProCA32.Collagen).

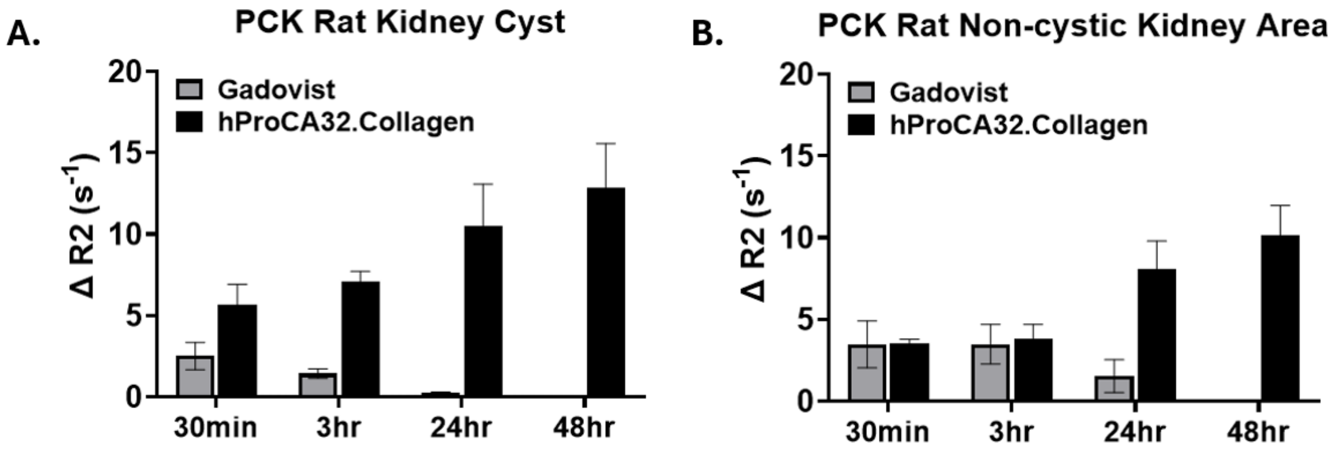

Figure S8. Longitudinal ΔR2 relaxometry in PCK rat kidney cysts and non-cystic parenchyma following hProCA32.Collagen or Gadovist administration. (A) Change in transverse relaxation rate (ΔR2, s^{-1}) measured longitudinally in PCK rat kidney cysts at 30 minutes, 3 hours, 24 hours, and 48 hours post-injection for hProCA32.Collagen (black bars, 0.010 mmol/kg) and Gadovist (gray bars, 0.10 mmol/kg). (B) ΔR2 measured in the non-cystic kidney parenchyma of PCK rats across the same time points. hProCA32.Collagen demonstrated progressive ΔR2 accumulation reaching approximately 10 s^{-1} at 48 hours. The sustained and progressive ΔR2 enhancement observed in both cystic and non-cystic compartments with hProCA32.Collagen confirms specific molecular retention within collagen-rich fibrotic microenvironments rather than passive fluid accumulation. Data are mean ± SEM. n = 8 PCK rats (hProCA32.Collagen); n = 5 PCK rats (Gadovist).

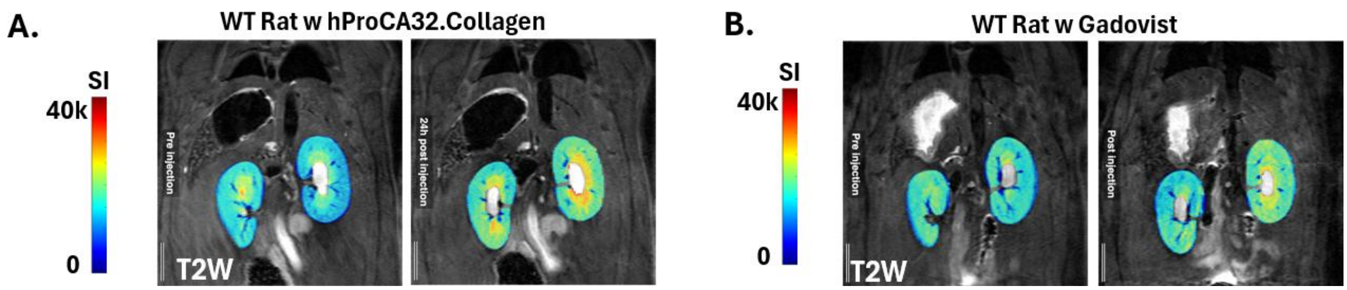

Figure S9. T2-weighted MRI signal intensity maps of WT rat kidneys pre- and post-injection with hProCA32.Collagen and Gadovist. (A) Representative T2-weighted MRI signal intensity (SI) colormaps of WT rat kidneys pre-injection and at 24 hours post-injection with hProCA32.Collagen, demonstrating moderate parenchymal signal enhancement at 24 hours consistent with renal clearance and nonspecific distribution in the absence of cystic or fibrotic pathology. (B) Representative T2-weighted MRI signal intensity colormaps of WT rat kidneys pre-injection and at 1 hour post-injection with Gadovist, demonstrating minimal and transient signal change at the peak enhancement timepoint, consistent with rapid renal clearance of the small-molecule gadolinium-based contrast agent in healthy kidney parenchyma. SI color scale: 0 (blue) to 40k (red).

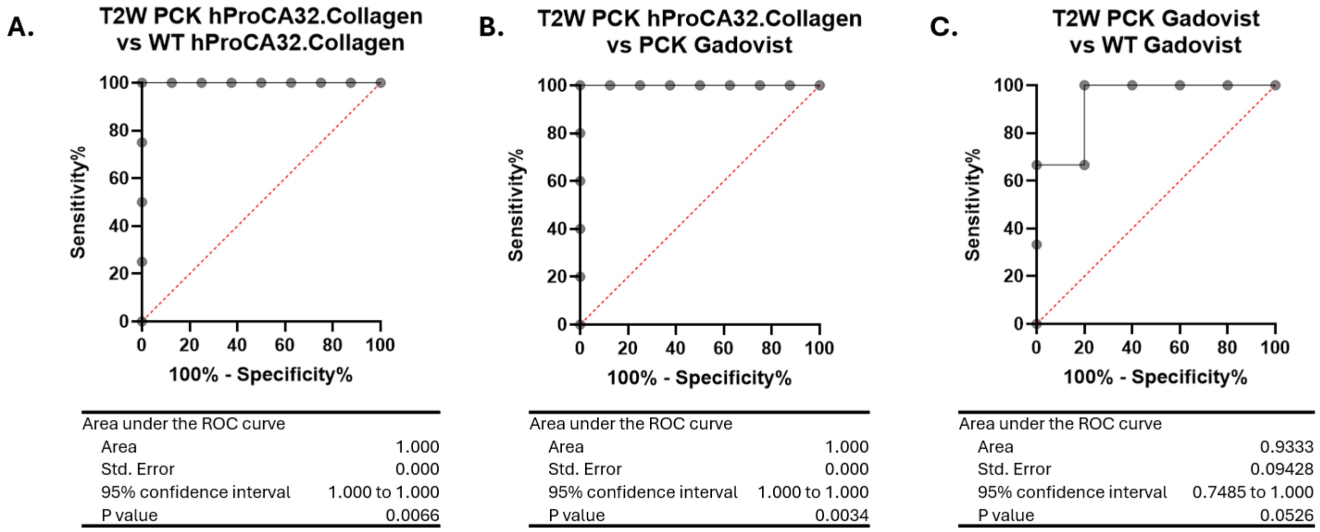

Figure S10. ROC curve analysis demonstrates perfect diagnostic accuracy of Gd-hProCA32.Collagen for discrimination of PCK fibrotic kidneys from controls using T2W MRI. (A) ROC curve for T2W SNR enhancement comparing PCK Gd-hProCA32.Collagen (24 hours post-injection) versus WT Gd-hProCA32.Collagen, demonstrating perfect discrimination of fibrotic from normal kidneys (AUC = 1.000, 95% CI: 1.000–1.000, p = 0.007). (B) ROC curve for T2W SNR enhancement comparing PCK Gd-hProCA32.Collagen (24 hours post-injection) versus PCK Gadovist (1 hour post-injection, peak enhancement timepoint), demonstrating perfect head-to-head superiority of collagen-targeted MRI over the clinical standard agent (AUC = 1.000, 95% CI: 1.000–1.000, p = 0.003). (C) ROC curve for T2W SNR enhancement comparing PCK Gadovist versus WT Gadovist at peak enhancement (1 hour post-injection), demonstrating non-significant discrimination between fibrotic and normal kidneys with the clinical standard agent (AUC = 0.933, 95% CI: 0.749–1.000, p = 0.053). SNR enhancement values were extracted at the peak enhancement timepoint for each agent, ensuring evaluation under optimal imaging conditions. The optimal diagnostic threshold was determined by Youden's J index. Red dashed line = chance level (AUC = 0.5).

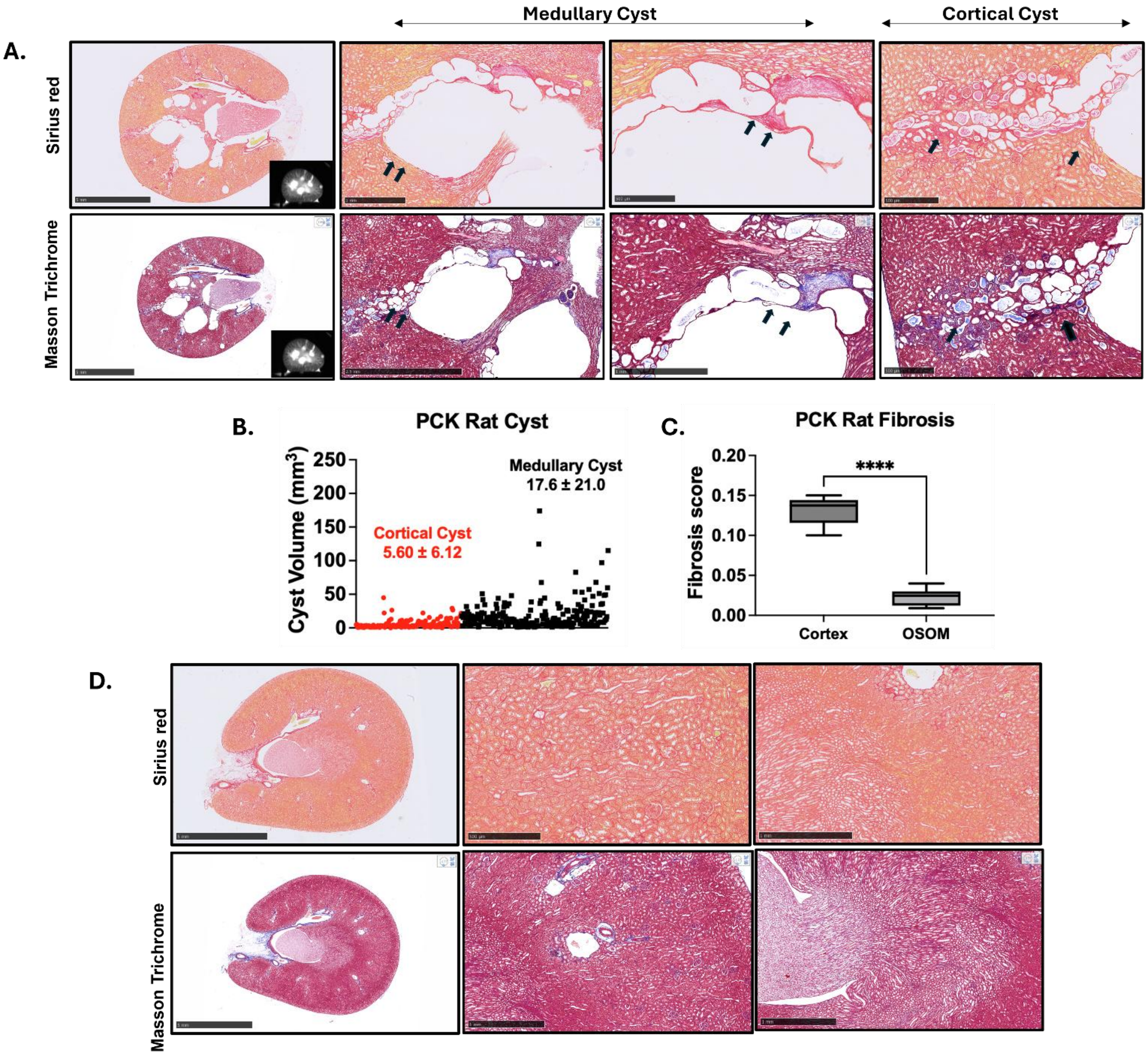

Figure S11. Region-specific fibrosis and cyst distribution in PCK rat kidneys. (A) Representative Sirius Red (top) and Masson's Trichrome (bottom) stained sections of PCK rat kidneys illustrating medullary (left columns) and cortical (right columns) cysts, with whole kidney low-magnification images and ex vivo MRI insets for spatial context. Arrows indicate pericystic and interstitial collagen deposition in both compartments. Scale bars shown. **(B)** Individual cyst volumes stratified by compartment demonstrating substantially larger medullary cysts relative to cortical cysts (17.6 ± 21.0 mm^3 vs. 5.60 ± 6.12 mm^3). **(C)** Quantitative fibrosis scoring revealing significantly greater fibrotic burden in the cortex relative to the OSOM (**** $p < 0.0001$), establishing spatially distinct pathological processes, cortex-predominant microfibrosis, and OSOM-predominant cyst expansion. **(D)** Representative Sirius Red (top) and Masson's Trichrome (bottom) stained kidney sections from wild-type control rats. Scale bars shown. Data are median with interquartile range. ****$p < 0.0001$.

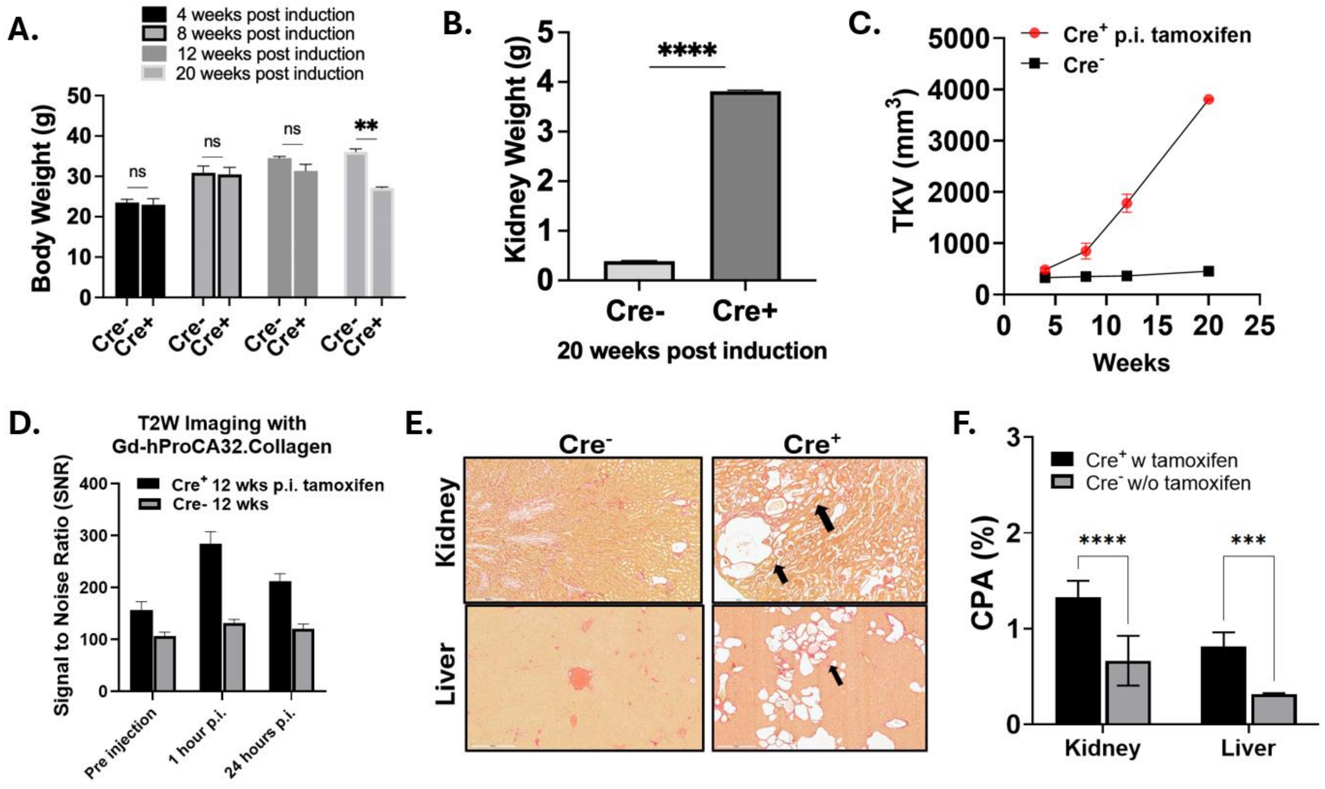

Figure S12. Longitudinal characterization and collagen-targeted MRI validation in a tamoxifen-inducible Cre^{+} mouse model of ADPKD. (A) Body weight comparison between Cre^{-} and Cre^{+} mice at 4, 8, 12, and 20 weeks post-induction. No significant differences were observed at 4, 8, or 12 weeks (ns); Cre^{+} mice showed significantly lower body weight at 20 weeks compared to Cre^{-} controls ($p < 0.01$), consistent with progressive systemic disease burden. (B) Kidney weight at 20 weeks post-induction demonstrating significantly greater kidney weight in Cre^{+} versus Cre- mice ($p < 0.0001$), reflecting extensive cyst-driven organ enlargement. (C) Longitudinal MRI-derived total kidney volume (TKV, mm^3) in Cre^{+} (tamoxifen p.i.) and Cre^{-} mice from 4 to 20 weeks post-induction, demonstrating exponential TKV growth in Cre^{+} mice reaching ~3,800 mm^3 by 20 weeks versus minimal change in Cre^{-} controls. (D) T2W MRI signal-to-noise ratio (SNR) at pre-injection, 1 hour, and 24 hours post-injection of Gd-hProCA32.Collagen in Cre^{+} (12 weeks post-induction) versus Cre^{-} mice, demonstrating significantly elevated and sustained enhancement in Cre^{+} mice at both timepoints. (E) Representative Sirius Red histological sections of kidney (top) and liver (bottom) from Cre^{-} (left) and Cre^{+} (right) mice, demonstrating progressive cyst formation and pericystic collagen deposition in Cre^{+} kidneys and liver (black arrows). (F) CPA quantification confirming significantly greater collagen deposition in Cre^{+} versus Cre- mice in both kidney ($p < 0.0001$) and liver ($p < 0.001$). Data points represent mean ± SEM.
